# A hospital-based child and adolescent overweight and obesity treatment protocol transferred into a community healthcare setting

**DOI:** 10.1371/journal.pone.0173033

**Published:** 2017-03-06

**Authors:** Pernille Maria Mollerup, Michael Gamborg, Cæcilie Trier, Christine Bøjsøe, Tenna Ruest Haarmark Nielsen, Jennifer Lyn Baker, Jens-Christian Holm

**Affiliations:** 1 The Children’s Obesity Clinic, Department of Pediatrics, Copenhagen University Hospital Holbæk, Holbæk, Denmark; 2 Institute of Preventive Medicine, Bispebjerg and Frederiksberg Hospital, The Capital Region, Frederiksberg, Denmark; 3 The Novo Nordisk Foundation Center for Basic Metabolic Research, Section for Metabolic Genetics, University of Copenhagen, Copenhagen, Denmark; 4 Faculty of Health and Medical Sciences, University of Copenhagen, Copenhagen, Denmark; Weill Cornell Medical College in Qatar, QATAR

## Abstract

**Background:**

Due to the pandemic of child and adolescent overweight and obesity, improvements in overweight and obesity treatment availability and accessibility are needed.

**Methods:**

In this prospective study, we investigated if reductions in body mass index (BMI) standard deviation scores (SDS) and waist circumference (WC) would occur during 1.5 years of community-based overweight and obesity treatment based upon an effective hospital-based overweight and obesity treatment protocol, The Children’s Obesity Clinics’ Treatment protocol. Height, weight, and WC were measured at all consultations. Changes in BMI SDS and WC were analyzed using linear mixed models based upon the repeated measures in each child.

**Results:**

From June 2012 to January 2015, 1,001 children (455 boys) were consecutively enrolled in the community-based treatment program. Upon entry, the median age was 11 years (range: 3−18), and the median BMI SDS was 2.85 (range: 1.26−8.96) in boys and 2.48 (range: 1.08−4.41) in girls. After 1.5 years of treatment BMI SDS was reduced in 74% of the children. BMI SDS was reduced by a mean of 0.38 (95% confidence interval (CI): 0.30−0.45, p<0.0001) in boys and 0.18 (95% CI: 0.12−0.25, p<0.0001) in girls after 1.5 years of treatment, independently of baseline age, BMI SDS, and Tanner stage (all p>0.08). WC was reduced by a mean of 3.8 cm (95% CI: 2.7−4.9, p>0.0001) in boys and 5.1 cm (95% CI: 4.0−6.2, p>0.0001) in girls. The dropout rate was 31% after 1.5 years. A median of 4.5 consultation hours was invested per child per year.

**Conclusion:**

BMI SDS and WC were reduced after 1.5 years of treatment. Hence, this community-based overweight and obesity treatment program may help accommodate the need for improvements in treatment availability and accessibility.

## Introduction

The prevalence of children and adolescents with overweight and obesity has reached alarmingly high levels worldwide [1]. In Denmark, the prevalence is 10% in preschool children [2] and 15 to 25% in adolescents [3]. Child and adolescent overweight and obesity is accompanied by co-morbidities [4] which lead to an increased risk of cardiovascular disease, diabetes, and premature death in adulthood [5,6]. Furthermore, children and adolescents with overweight and obesity are frequently burdened by psychosocial problems, and their quality of life is compromised [7]. Community-based treatment options have the advantages of being situated in the families’ local area and being accessible by self-referral, and thus may help improve treatment availability and accessibility [8,9]. Nonetheless, few overweight and obesity treatment programs have been evaluated in community healthcare settings, the results are mixed, and the long-term treatment effects are unclear [8–14]. In the United Kingdom, a nine-week community-based obesity treatment of 116 children aged 8−12 years resulted in a 0.30 reduction in body mass index (BMI) standard deviation score (SDS) (p<0.001), but at a 2.4-year follow-up after the MEND program, BMI SDS was not lower than at entry [14]. In America, a six-months community-based obesity treatment of 155 children and adolescents aged 6−17 years resulted in only a 0.06 reduction in BMI SDS (p<0.001) [12,13]. Consequently, the feasibility of community-based treatment of children and adolescents with overweight and obesity has been questioned [8].

An evidence-based chronic care, overweight and obesity treatment program titled The Children’s Obesity Clinic’s Treatment (TCOCT) protocol has been developed at The Children’s Obesity Clinic at the Pediatric Department, Copenhagen University Hospital Holbæk [15]. Since 2008, over 2,300 children and adolescents with overweight and obesity, aged 0−24 years have been consecutively enrolled in the treatment program. In 2011, reductions of 0.40 BMI SDS in boys and 0.25 BMI SDS in girls after 1.5 years of treatment were documented [15]. Importantly, several co-morbidities were reduced as well [16–18]. Similar reductions in BMI SDS were documented when the TCOCT protocol was transferred to another Danish pediatric hospital department [19]. However, if reductions in BMI SDS will occur, when the TCOCT protocol is transferred into a community healthcare setting, has yet to be evaluated.

Therefore, we investigated if reductions in BMI SDS and waist circumference (WC) would occur when the TCOCT protocol was transferred into a chronic care, child and adolescent overweight and obesity treatment program situated at community healthcare centers across Denmark, during 1.5 years of treatment.

## Materials and methods

### Initiation of the community-based treatment program

In Denmark, community healthcare is provided at healthcare centers situated in each municipality. These centers provide services such as preventive child health examinations, and services to children and families with special needs. Nonetheless, no community-based standard child and adolescent overweight and obesity treatment program exists in Denmark.

Participation of the healthcare centers in this study was decided by the healthcare management in each municipality and thus, the participating centers were not randomly selected. To be eligible to participate, the healthcare centers were required to allocate the necessary equipment, personnel (nurses and dietitians), and time for training in the TCOCT protocol. The nurses were required to have specialized in the care of children and the dietitians had to be certified clinical dietitians or have a bachelor degree in nutrition and health. The nurses and dietitians’ training consisted of initial theoretical courses (three days) and follow-up courses (four days), taught by experienced personnel from The Children’s Obesity Clinic, and an internship at The Children’s Obesity Clinic (seven to nine days). During this internship, the community personnel attended consultations conducted by pediatricians, dietitians, and nurses at The Children’s Obesity Clinic. Further, all community healthcare professionals received two days of supervision of consultations at the local healthcare centers from an experienced nurse from The Children’s Obesity Clinic. The healthcare management in each municipality agreed to treat approximately 100 children and adolescents during the study period, so that the personnel delivering the treatment program would achieve and maintain appropriate routine and experience in using the TCOCT protocol during the study period. Finally, the healthcare management in each municipality were to allocate an average of six consultation hours for treatment per child or adolescent per year [15].

The healthcare managements in eight municipalities agreed to participate in the study. Thus, from February 2012 to April 2013, child and adolescent overweight and obesity treatment programs based upon the TCOCT protocol were established at eight community healthcare centers.

### Sample size

To estimate the required size for the study population, a power calculation was conducted. The minimal relevant difference in BMI SDS was defined as 0.10, as a reduction of this size has previously been associated with health benefits [20]. A standard deviation on the change in BMI SDS of 0.45 was derived from data from The Children’s Obesity Clinic. Thus, with a power of 0.90, a significance level of 0.05, and a two-sided paired t-test, we found that the required size of the study population was 215 children. To account for a 30% dropout, a study population of 307 children was required. However, as no community-based standard care treatment program existed, and as lifestyle interventions such as the TCOCT program are unlikely to cause any harm [21], an overweight and obesity treatment program based upon the TCOCT protocol was initiated in all municipalities that volunteered for this. Changes in BMI SDS and WC during the community-based treatment programs established were evaluated in the present study.

### Patients

**Fig 1:**
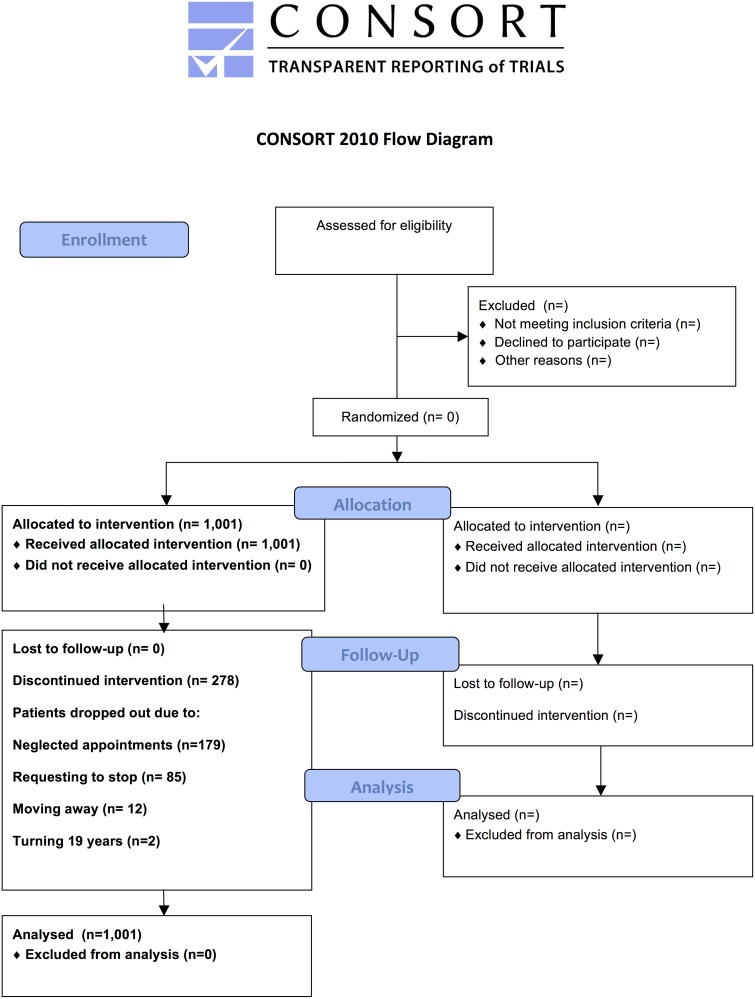
CONSORT diagram.

The consecutive enrolment at the centers began on June 7^th^, 2012 and is still ongoing (Fig 1). Information about the availability of the treatment program was made public on the healthcare center’s websites and through leaflets at these centers. Children and adolescents were referred to the treatment by general practitioners, school nurses, or directly by their families. This study included all children and adolescents enrolled at the centers until January 23^rd^, 2015, and follow-up ended on March 14^th^, 2015. The criteria for entering into treatment were an age from 3−18 years and a BMI equal to or above the 85^th^ percentile according to Danish age- and sex-specific references (equivalent to a BMI SDS of 1.04) [22]. There were no other eligibility criteria for entering into treatment.

### The community-based treatment program

The TCOCT protocol is described elsewhere [15]. The theoretical framework is that obesity is regarded as a complex and chronic disease [23] due to the tight neuroendocrine regulation of fat mass counteracting attempts at weight loss in patients with overweight and obesity [24] and to the multitude of medical [4] and psychosocial complications [7]. Therefore it requires a comprehensive and chronic care treatment [23].

**Fig 2:**
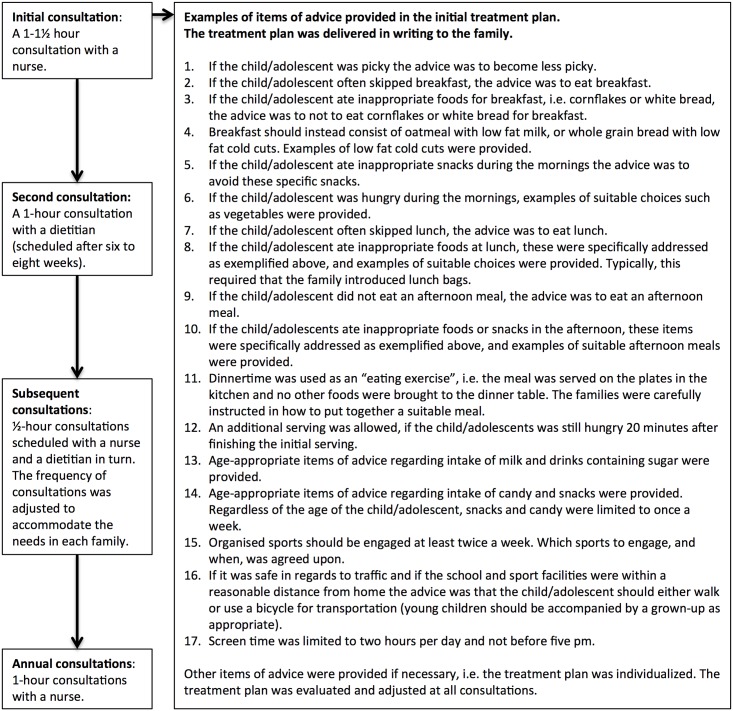
The community-based treatment program.

All consultations were one-to-one with each family and the treatment program began with a thorough questionnaire-based interview aimed at identifying lifestyle changes needed to optimize the child’s daily life to achieve weight loss (S1 File) [25–27]. These lifestyle changes were incorporated into an individualized treatment plan for the family (Fig 2). All families consulted with a nurse initially and approximately annually thereafter, and with a dietitian four to eight weeks after the initial consultation. In-between consultations were scheduled with a nurse and dietitian in turn. The frequency of in-between consultations was agreed upon by the families and the healthcare professionals and was adjusted to accommodate the needs and challenges in each family. Thus, consultations were scheduled more frequently with families experiencing difficulties in implementing the lifestyle changes than with families without these difficulties. As the challenges within a family may change over time, the frequency of consultations often varied over time as well. The treatment continued until normal weight was achieved (BMI below the 85^th^ percentile [22,28]), until the child turned 19 years, or until the families requested to stop or repeatedly neglected appointments (more than two consecutive appointments or no response to a letter with an invitation to make a new appointment instead of the neglected consultation).

### Anthropometric measurements

Height, weight, hip circumference (HC), and WC were measured once at each consultation and were recorded by nurses or dietitians. Height was measured to the nearest 0.1 cm on a Tanita^®^ HR100 (Tanita Corp. Tokyo, Japan) stadiometer and weight to the nearest 0.1 kilogram on a Tanita^®^ BC418 scale (Tanita Corp. Tokyo, Japan). WC was measured post-exhalation at the level of the umbilicus [29]. HC was measured at the level yielding the widest distance around the buttocks [30]. Measurements were conducted with the child or adolescent wearing light indoor clothes and without shoes.

### Socioeconomic status

Socioeconomic status was categorized into five groups based upon parental occupation, using the Statistics Denmark’s national classification, with one being the highest and five the lowest [31].

### Pubertal development stage

Upon entry, the Tanner pubertal development stage was self-reported in a questionnaire. Self-assessment was guided by written instructions and a depiction of pubertal development stages [32,33]. In girls, questions regarding the time of menarche were asked, and if it had occurred, the girl was considered pubertal. If the questionnaire was not completed, girls younger than eight years and boys younger than nine years of age were considered pre-pubertal [34].

### Ethical considerations

All children and adolescents gave informed consent and their parents signed a written consent upon entry. The study was approved by the regional Danish Ethics Committee (January 26^th^, 2009, protocol ID SJ-104) and the Danish Data Protection Agency. The study was initially registered as a part of the research activities in the “The Danish Childhood Obesity Biobank” (Clinicaltrials.gov, ID no.: NCT00928473). However, during study process, we found it more appropriate to register the present study independently. Clinicaltrials.gov ID no.: NCT02013843. The authors confirm that all ongoing and related trials for this intervention are registered.

### Statistics

Statistics were performed using R statistical software version 3.2.2 (http://www.r-project.org) and SAS software version 9.4 (SAS Institute Inc., Cary, North Carolina, USA). BMI was converted into a BMI SDS using the LMS method based on Danish sex- and age-specific BMI references [22,35]. Baseline age, BMI SDS, HC, WC, socioeconomic status, and the duration of treatment were compared in boys and girls using the Wilcoxon signed rank test. Percentages of children and adolescents with overweight or obesity, socioeconomic status, region of residence, and Tanner stages were compared in boys and girls using the Chi-squared test.

The treatment time was calculated as the time from the first consultation until the study period ended or until dropout. Since previous studies using the TCOCT protocol, have found that changes in BMI SDS differ between boys and girls [15,19], we performed separate analyses in boys and girls. Due to the consecutive enrolment of children into treatment, the treatment time varied between the children. Due to the individualized treatment program, the number of measures of height and weight in each child varied as well the time between the measurements. Further, other studies using the TCOCT protocol [15,19] have found non-linear reductions in BMI SDS during the time course of treatment. To accommodate these considerations, we used linear mixed models to analyze the changes in BMI SDS and WC [15,19]. These models were based upon all measures in all children during this study period. However, as only 5.6% of the measurements were beyond 18 months of treatment, we found the data to be inadequate to model the changes beyond 18 months of treatment. In the linear mixed models, changes in the mean values of BMI SDS and WC were modeled as functions of time. The models had a covariance structure with a random intercept and slope and an exponential residual structure. These models allowed for individual levels and the development of BMI SDS or WC (random effects) and for the covariance between two measurements on the same child to decrease as time between the measurements increased [15,19]. Healthcare center was included as a random effect. The fixed effects were time, BMI SDS upon entry, socioeconomic status, and Tanner stage. Time was included as a cubic spline with three a priori cut-points at the 10th, 50th, and 90th percentiles, corresponding to 2, 7, and 17 months of treatment, respectively. The change in WC was adjusted for age, so that we compared the average WC before and after treatment for a given age. Thus, we compared the WC in children after for example one year of treatment with the WC in children with the same age when entering into treatment (e.g. comparing the WC in 11 year old boys after a year of treatment (starting the treatment at age 10 years) with the WC in 11 year old boys entering into treatment). Associations of baseline age, BMI SDS, socioeconomic status, and Tanner stage with changes in BMI SDS were analyzed by testing for an interaction between dichotomized baseline characteristics and time since enrolment. The dichotomized baseline characteristics were defined as follows: younger or older than the median age, BMI SDS below or above three SDS, high socioeconomic status (1−3) or low socioeconomic status (4−5), pre-pubertal (Tanner 1) or pubertal and post-pubertal (2−5). Percentages of children reducing BMI SDS were calculated using the latest measurements in the children and adolescents still enrolled after 3, 6, 9, 12, 15, or 18 months of treatment.

To supplement the results of the linear mixed models, we performed simple pre-post analyses of the changes in BMI SDS and WC from the initial to the latest consultation attended within the study period. The paired t-test was used for these analyses.

Due to the consecutive enrolment of children into treatment during the study period, the dropout rates after 1 year and 1.5 years were calculated using the Kaplan-Meier estimates. Children who were ineligible after 1 and 1.5 years of treatment due to late enrolment were censured in these analyses. Associations of the dropout rate with sex and dichotomized age, BMI SDS, socioeconomic status, and Tanner stage were analyzed in Cox regression models. Changes in mean values of BMI SDS before dropout were analyzed using the paired t-tests comparing BMI SDS upon entry into treatment with BMI SDS at the latest consultation before dropout. Using the independent t-test, reductions in BMI SDS at the latest consultation attended were compared in the children who dropped out with the children who remained in treatment during the study period.

Differences in sex, age, BMI SDS, socioeconomic status, and Tanner stage at the healthcare centers were analyzed using the Kruskal-Wallis rank sum test and the Chi-squared test. In the linear mixed models, associations of healthcare center with reductions in BMI SDS were analyzed by testing for interactions between a variable comparing the rate of BMI SDS reduction at each center to the center with the highest rate of BMI SDS reduction and the times since enrolment. These analyses were supplemented by an overall test (Kruskal Wallis test) of the difference in mean changes in BMI SDS at the latest consultation in children enrolled at each center. Further, analyses of the mean changes in BMI SDS upon the latest consultation in boys and girls at each healthcare center were performed using the paired t-test. Dropout rates at the clinics were calculated using the Kaplan-Meier estimates, and association of healthcare center with dropout rate was analyzed using Cox-regression analysis adjusted for baseline characteristics.

To analyze the association between the frequency of consultations and the BMI SDS reduction rate, the median number of consultations per year was calculated in children enrolled for more than one year. Subsequently, the associations with changes in BMI SDS were analyzed by testing for interactions comparing the rate of BMI SDS change in children with frequent (more than the median number per year) or infrequent consultations.

## Results

### Baseline

During the study period 1,001 children and adolescents (455 boys) entered treatment. 92.8% were Caucasian, 5.6% were of Middle Eastern origin, and 1.4% were of other origins (Asian, African, Inuit, Hispanic, or other). The median age upon entry was 10.9 years in boys and 10.8 years in girls (Table 1). The median BMI SDS was 2.85 in boys and 2.48 in girls.

**Table 1:** Characteristics of the study population.

	Boys	Girls	P-value
**N** (%)	455 (45.5)	546 (54.5)	
**Age** (years, median−range)	10.9 (3.0−18.3)	10.8 (3.2−18.0)	0.58
**BMI SDS** (median−range)	2.85 (1.26−8.96)	2.48 (1.08−4.41)	<0.001
Overweight* (n, %)	20 (4.4)	34 (6.2)	0.26
Obese** (n, %)	435 (95.6)	512 (93.8)	
**WC** (cm, median−range)	86.8 (58.0−143.0)	85.0 (54.0−134.5)	0.16
**HC** (cm, median−range)	92.0 (48.5−148.0)	92.0 (54.0−141.0)	0.40
**Tanner stage** (median−range)	2 (1−5)	3 (1−5)	<0.001
Pre-pubertal (n, %)	112 (36.5)	98 (21.0)	<0.001
Pubertal or post-pubertal (n, %)	195 (63.5)	368 (79.0)	
**Socioeconomic status** (median−range)	3 (1−5)	3 (1−5)	0.18
High (n, %)	272 (67.5)	318 (64.0)	0.30
Low (n, %)	131 (32.5)	179 (36.0)	
**Region of residence**			
Region Zealand (n, %)	205 (45.1)	251 (46.0)	0.32
Region Mid Jutland (n, %)	104 (22.9)	119 (21.8)	
Region South Jutland (n, %)	146 (32.1)	176 (32.2)	

Data is presented as medians with ranges or as numbers and percentages.

BMI, body mass index; SDS, standard deviation score; N, number; WC, waist circumference; HC, hip circumference.

* Overweight: 85^th^−94^th^ percentile.

** Obese: ≥95^th^ percentile. Continuous variables were compared in boys and girls using the Wilcoxon signed rank test. Categorical variables were compared using the Chi-squared test.

### Treatment time

The median treatment time was 15 months (range 1−34 months) in both boys and girls. The median time from the initial to the latest consultation within the study period was 11 months (range: 0−30 months) in boys and girls. A total of 907 children (407 boys) attended at least two consultations, and in this group of boys and girls, the median time from the initial to the latest consultation was 12 months (range: 1−30 months).

### Changes in BMI SDS and WC

In the linear mixed models, we found that after 1 year of treatment, BMI SDS was reduced by a mean of 0.34 in boys and 0.22 in girls (p<0.0001; Table 2). After 1.5 years of treatment, BMI SDS was reduced by a mean of 0.38 in boys and 0.18 in girls. Among boys, 73.9% had reduced their BMI SDS and 50.8% had reduced their BMI SDS by more than 0.25 after 1.5 years of treatment. Among girls, 73.6% had reduced their BMI SDS and 44.4% had reduced their BMI SDS by more than 0.25 after 1.5 years of treatment. In both sexes, reductions in BMI SDS were independent of baseline age (boys: p = 0.08, girls: p = 0.54), BMI SDS (boys: p = 0.87, girls: p = 0.48), and pubertal development stage (boys: p = 0.18, girls = 0.87). Boys with low socioeconomic status attained a 0.11 smaller reduction in BMI SDS per year than boys with high socioeconomic status (95% CI: 0.005−0.22, p = 0.04), whereas girls reduced their BMI SDS independently of socioeconomic status (p = 0.82). After 1.5 years of treatment, mean reductions in WC were 3.8 cm in boys and 5.1 cm in girls (p<0.0001; Table 2).

**Table 2:** Changes in BMI SDS and WC during 1.5 years of treatment.

	3 months Estimate (95% CI)	6 months Estimate (95% CI)	9 months Estimate (95% CI)	12 months Estimate (95% CI)	15 months Estimate (95% CI)	18 months Estimate (95% CI)
**Δ BMI SDS**						
Boys	−0.23*	−0.30*	−0.32*	−0.34*	−0.35*	−0.38*
	(−0.20 to −0.26)	(−0.26 to −0.33)	(−0.28 to −0.37)	(−0.29 to −0.39)	(−0.29 to −0.41)	(−0.30 to −0.45)
Girls	−0.18*	−0.24*	−0.25*	−0.22*	−0.20*	−0.18*
	(−0.16 to −0.20)	(−0.22 to −0.27)	(−0.22 to −0.28)	(−0.18 to −0.26)	(−0.14 to −0.25)	(−0.12 to −0.24)
**Δ WC** (cm)						
Boys	−2.7*	−3.4*	−3.5*	−3.6*	−3.7*	−3.8*
	(−1.4 to −2.0)	(−2.9 to −3.9)	(−2.9 to −4.1)	(−2.8 to −4.3)	(−2.8 to −4.6)	(−2.7 to −4.9)
Girls	−3.4*	−4.2*	−4.3*	−4.4*	−4.6*	−5.1*
	(−3.0 to −3.8)	(−3.7 to −4.6)	(−3.7 to −4.9)	(−3.7 to −5.1)	(−3.7 to −5.5)	(−4.0 to −6.2)
**ΔBMI SDS groups**						
Boys	**(n = 318)**	**(n = 375)**	**(n = 334)**	**(n = 283)**	**(n = 208)**	**(n = 134)**
< −0.50	12.3%	25.1%	27.0%	29.0%	33.2%	32.1%
< −0.25 to −0.50	23.9%	20.8%	21.0%	23.7%	19.2%	18.7%
< 0.00 to −0.25	39.6%	32.0%	30.5%	26.1%	22.6%	23.1%
≥ 0.00	24.2%	22.1%	21.5%	21.2%	25.0%	26.1%
Girls	**(n = 397)**	**(n = 453)**	**(n = 420)**	**(n = 352)**	**(n = 264)**	**(n = 178)**
< −0.50	4.5%	15.0%	20.7%	23.6%	26.9%	29.2%
< −0.25 to −0.50	20.2%	23.2%	23.1%	21.0%	18.0%	15.2%
< 0.00 to −0.25	53.1%	41.0%	35.5%	30.1%	32.0%	29.2%
≥ 0.00	22.2%	20.8%	20.7%	25.3%	23.1%	26.4%

BMI, body mass index; SDS, standard deviation score; WC, waist circumference (age-adjusted); CI, confidence interval; n, number of boys/girls in the analyses.

* Statistically significant change in BMI SDS as a function of time (p<0.0001). Changes in BMI SDS and age-adjusted WC were analyzed using linear mixed models. Cumulative percentages with BMI SDS reductions were calculated in the children and adolescents who were eligible at each time point.

In the pre-post analyses, we found that in children who attended at least two consultations during the study period (91%), the mean reduction in BMI SDS at the latest consultation was 0.33 (95% CI: 0.28−0.38, p<0.0001) in boys and 0.22 (95% CI: 0.10−0.26, p<0.0001) in girls. The mean reduction in WC was 0.1 cm (95% CI: −0.6 to 0.8, p = 0.72) in boys and 1.3 cm (95% CI: 0.6−2.0, p = 0.0002) in girls.

### Dropout

During the study period of 34 months, 278 children and adolescents (123 boys) dropped out after a median of 9 months (range: 1−26). The participants dropped out due to neglected appointments (n = 179, 64.9%), requesting to stop (n = 85, 30.8%), moving away (n = 12, 4.3%), or turning 19 years of age (n = 2).

Of the children and adolescents who dropped out, 201 (73.0%) children attended at least two consultations before dropping out. In these children, BMI SDS was reduced by a mean of 0.16 (95% CI: 0.10−0.21, p<0.0001) at the latest consultation before dropout. This reduction in BMI SDS was smaller than the mean reduction in BMI SDS among the children who remained in treatment (p<0.0001). Of the children who dropped out, 11 children (4.0%) had achieved normal weight before dropping out. A total of 131 children (47.5%) had reduced their BMI SDS, and in this group the BMI SDS was reduced by a mean of 0.33 (95% CI: 0.27−0.39, p<0.0001). Seventy children (25.4%) had increased their BMI SDS before dropping out, and in this group the BMI SDS increased by a mean of 0.17 BMI SDS (95% CI: 0.12−0.22, p<0.0001).

The dropout rates were 19.8% (95% CI: 17.2−22.3) and 30.7% (95% CI: 27.4−33.9) after one and 1.5 years of treatment, respectively. Older children (age above the median) were more likely to drop out than younger children (hazard ratio: 1.56, 95% CI: 1.11−2.21, p = 0.01). Sex (p = 0.75), BMI SDS (p = 0.80), socioeconomic status (p = 0.60), and Tanner stage (p = 0.81) were not associated with the dropout rate.

### Time investments

In children and adolescents treated for more than one year, a median of 4.5 consultation hours (range: 1−9) was invested in the treatment of each child or adolescent per year. No differences were found in BMI SDS reduction rates in the children and adolescents attending consultations frequently (more than 4.5 consultations per year) or infrequently (boys: p = 0.34, girls: p = 0.38).

### Healthcare centers

A median of 121 children and adolescents (range: 83−196) entered treatment at each center. The median age, BMI SDS, and Tanner stage (girls) differed at the centers (p<0.05), whereas sex, socioeconomic status, and Tanner stage (boys) did not differ (p>0.20).

In the linear mixed models, we found an interaction between BMI SDS reduction rate and healthcare center. Thus, at the centers with the highest reduction rates, BMI SDS was reduced by 0.26 (CI 95%: 0.10−0.42, p = 0.002) more in boys and by 0.18 BMI SDS (CI 95%: 0.04−0.33, p = 0.01) more in girls after one year than at the centers with the lowest reduction rates.

In pre-post analyses, we found an overall difference in mean reductions in BMI SDS at the healthcare centers among boys (p = 0.02) and girls (p = 0.001). The mean reductions in BMI SDS at the latest consultation ranged from 0.19 (95% CI: 0.05−0.33, p = 0.01) to 0.45 (95% CI: 0.25−0.66, p<0.0001) in boys and from 0.12 (95% CI: 0.02−0.22, p = 0.02) to 0.31 (95% CI: 0.21−0.42, p<0.0001) in girls. The dropout rates differed at the centers (p<0.0001), and ranged from 2.5% to 26.5% after one year, and from 13.2% to 42.8% after 1.5 years.

## Discussion

BMI SDS and WC were reduced in boys and girls during 1.5 years of community-based child and adolescent overweight and obesity treatment based upon the TCOCT protocol [15]. Although not directly compared in this study, the reductions in BMI SDS achieved in this community healthcare setting were at a level similar to those reported in the evaluations of the TCOCT protocol delivered at two hospital pediatric departments. The results of these evaluations were based upon the same statistical models as were used in the present study [15,19]. Further the reductions in BMI SDS were at a level similar to the initial reductions reported in the evaluation of another community-based childhood obesity treatment program [11]. Thus, these results support that treating child and adolescent overweight and obesity in a community healthcare setting is feasible.

Weight regain is often reported after overweight and obesity treatment [14,28]. In this study, the mean BMI SDS declined throughout 1.5 years of treatment in boys. In girls, the mean BMI SDS increased slightly from 9 to 18 months, but it did not increase to the level upon entry. Hence, this chronic care treatment approach may be helpful in preventing weight regain [23,27].

In this study, 51% of the boys and 44% of the girls attained a reduction in BMI SDS greater than 0.25. Other studies, including those using the TCOCT protocol, have shown that reductions in BMI SDS of 0.25 or more are associated with improvements in components of the metabolic syndrome [15–18,36], indicating that the BMI SDS reductions achieved in this study were at a level that is likely to reduce cardiovascular disease risk factors [36].

Consistent with previous studies using the TCOCT protocol [15,19], we found different reductions in BMI SDS between boys and girls. In 2011, a Cochrane review found no evidence supporting that patterns of weight loss differ by sex. Although this difference has been consistent in evaluations of the TCOCT protocol, it is unclear as to why it occurs, and further research is needed to elucidate the reasons for this finding.

A number of studies have reported difficulties in treating adolescents with obesity [37]. We found that weight loss was independent of age, but that older children were more likely to drop out, thus highlighting the need of management in this group. Overweight and obesity are prevalent in families with low socioeconomic status, and these families may constitute a potentially underserved group [38]. Thus, providing professional service to these families is important [38]. In our study, the BMI SDS was reduced independently of socioeconomic status in girls, whereas boys with low socioeconomic status attained slightly smaller reductions in the BMI SDS. Importantly, due to the particularly large reductions of BMI SDS in boys, the reductions were still considerable even in the boys with low socioeconomic status.

### Dropout

Traditionally, overweight and obesity treatment programs are complicated by dropout rates as high as 83% [9,39]. The overall 20% dropout rate after one year of treatment is consistent with the 10% [19] and 24% [15] reported in the evaluations of the TCOCT protocol delivered at hospital pediatric departments. In another community-based childhood obesity treatment program [11], a 10% dropout during the treatment program was reported. However, this program lasted only nine weeks, and 38% of the participants did not attend the six-month follow-up [11]. Thus, a higher dropout may be expected during a longer treatment program, and comparing treatment programs with different lengths may not be meaningful. We found that smaller reductions in BMI SDS occurred in children who dropped out compared with the reductions in BMI SDS in the children who remained in treatment. Nonetheless, 48% of the children dropping out reduced their BMI SDS before dropping out, and 4% achieved a normal weight. Hence, dropping out may not always be due to failure in reducing the BMI SDS.

### Time investments

The 4.5 consultation hours invested in treating each child or adolescent per year in our study is consistent with the 4.5 hours [19] and 5.4 hours [15] reported in the evaluations of the TCOCT protocol delivered at hospital pediatric departments. Few studies report the exact time investments of treatment programs and comparing treatment programs is complicated by differences in the content of one-to-one sessions and group sessions with varying numbers of children attending.

Intensive programs are often more effective [40]. In this study, frequent consultations (more than 4.5 per year) were not associated with greater reductions in BMI SDS than infrequent consultations. However, the number of consultations was individualized and frequent consultations were scheduled with families having difficulties in implementing or maintaining the lifestyle changes, whereas fewer consultations were scheduled with families with fewer difficulties. Therefore, we cannot conclude that frequent or infrequent consultations are preferable. That considerable reductions in BMI SDS occurred independently of whether greater or less than 4.5 consultation hours were held may suggest that an appropriate number of consultations were provided in this individualized treatment modality. Alternately, it is possible that BMI SDS was reduced, as those who chose to enroll were motivated and would have reduced their weight even if they were not treated. Nonetheless, this may not be the most plausible explanation as other studies have found only small reductions in BMI SDS in children who were on waiting lists and thus were equally motivated but received no treatment [11,41].

### Healthcare centers

The reductions in BMI SDS and the dropout rates differed at the healthcare centers, despite equal and thorough supervision of the personnel. There are several plausible reasons for this: all community healthcare professionals had relevant educations and were considered qualified to deliver the treatment. Nonetheless, variations in the skills of the personnel may still have differed. Equally important, the patients enrolled at each healthcare center may have differed. Due to the complexity of obesity and the predisposition to obesity [42], some families may have experienced greater challenges in attaining or maintaining weight loss during treatment than others. Further, the BMI SDS reduction rates changed over time, and at any given time, such differences may occur, regardless of overall similar reduction rates. And overall, BMI SDS was reduced, and at the latest consultation, BMI SDS was reduced by at least 0.19 in boys and 0.12 in girls at all healthcare centers. Similarly, the dropout rates did not exceed 26.5% after one year at any healthcare center.

### Strengths and limitations

The strengths of the present study are the relatively large number of children and adolescents included in the study and the relatively long period of treatment. Considerable efforts were invested in optimizing the personnel’s adherence to the treatment protocol; all nurses and dietitians were thoroughly trained and follow-up lessons and local supervision was conducted throughout the study period. The personnel at The Children’s Obesity Clinic were highly experienced in using the TCOCT protocol but were relatively inexperienced in transferring the treatment protocol into a community healthcare setting. Therefore, investing a considerable amount of time in the training of the community personnel was prioritized. However, with the experience acquired while conducting this study, providing a more effective and thus less time consuming initial training may be feasible.

We have no information on the children and adolescents that did not participate in the treatment program, or on why some families neglected appointments or requested to end treatment. We did not follow the children who dropped out, and thus we have no information on the treatment effect after dropout. Addressing this is an important challenge.

In this community healthcare setting with limited equipment available, we evaluated the changes in BMI SDS and WC. These anthropometric measures may not capture all health changes during overweight and obesity treatment [43], and we do not know if cardiovascular disease risk factors were reduced during treatment as well. Nonetheless, in children and adolescents with overweight or obesity, BMI SDS and WC correlate well with fat mass and with the cardiovascular risk burden [5,29,44]. Therefore, BMI SDS and WC are useful measures in healthcare settings such as this without advanced equipment. We did not calculate WC SDS. Instead, we adjusted for age and provided sex-specific results. The pre-post analyses of changes in WC were not adjusted for age, which may explain why the reductions in WC in boys were not statistically significant in these analyses.

Due to variations in treatment time and frequency of consultations, and due to non-linear reductions in BMI SDS, we used linear mixed models that were based upon all measurements performed in all children, to describe the changes in BMI SDS and WC during the time course of the treatment program. The results of these analyses were supported by the results of simple pre-post analyses.

The pubertal development stage was self-reported using a non-validated questionnaire, and this method may be less accurate than a physical examination by an experienced pediatrician [45]. However, as pediatricians were not employed at the healthcare centers this was not possible.

The study was observational, and not a randomized controlled study. Thus, we cannot conclude that the reductions in BMI SDS were due to the treatment *per se*. The study design choice was motivated by the lack of community-based standard care treatment for children and adolescents with overweight or obesity in Denmark; therefore we found it unethical to randomize these children to no treatment or to a waiting list [46]. Studies including control groups assigned to minimal or no-treatment often report little or no reductions in BMI SDS. Thus, these children are not helped [47]. Instead, a randomized controlled study could be conducted by randomizing children for treatment delivered at a hospital pediatric department or in a community healthcare setting. However, for practical and economic reasons, this was not feasible at the time of initiation of this community-based treatment program.

Traditionally, childhood obesity treatment programs have been evaluated in “ideal” controlled academic settings using numerous exclusion criteria to ensure uniform and comparable patient groups. There are obvious scientific advantages to this, but it also compromises the applicability in the clinical reality [8]. In this study, we evaluated a community-based treatment program setup to continue past the study period, and without prior exclusion of children or adolescents. Thus, the study represents the actual clinical reality. As of November 2016, the community-based TCOCT program was still ongoing at all healthcare centers, suggesting that the program is affordable, realistic, and was well received at the healthcare centers. The healthcare centers were not randomly selected and those participating in the study may be more committed to providing this treatment program than others. However, participation in the study was decided by the healthcare managements and not by the personnel delivering the treatment.

## Conclusions

During this prospective and observational study, we found that clinically meaningful reductions in BMI SDS occurred in both sexes, regardless of baseline age, BMI SDS, and stage of pubertal development when the TCOCT protocol was transferred to a community healthcare setting. The treatment program was still ongoing at all healthcare centers more than one year after the end of this study period. Thus, while recognizing the limitations of the non-randomized study design, the results of this study suggest that this community-based overweight and obesity treatment program is feasible and can be effective, and thus may provide a viable model to help accommodate the need for improvements in child and adolescent overweight and obesity treatment availability and accessibility. The study was executed in Denmark, and the TCOCT protocol was developed at a Danish pediatric department. However, the principles of the TCOCT protocol are not specific to Denmark and could potentially be transferred to hospital or community healthcare settings in other countries.

## Supporting information

S1 File English translation of the questionnaire used at the initial consultation. (DOC)

S2 File Study protocol—English version. (DOCX)

S3 File Study protocol—Danish version. (DOCX)

S4 File TREND checklist. (PDF)

S5 File Dataset. (CSV)

## Abbreviations

BMI: Body mass index
HC: Hip circumference
SDS: Standard deviation score
TCOCT: The Children’s Obesity Clinic’s Treatment
WC: Waist circumference
