# Supplementary material for: A hospital-based child and adolescent overweight and obesity treatment protocol transferred into a community healthcare setting
Source: PLoS One. 2017 Mar 6;12(3):e0173033. doi: 10.1371/journal.pone.0173033 (PMC5338817; doi:10.1371/journal.pone.0173033)
Supplement: S1 File — (DOC) [file pone.0173033.s001.doc]

**Questionnaire – initial consultation**

**The Children’s Obesity Clinic’s Treatment protocol**

**- Community-based treatment**

**ID:**

______ -year old__________ is seen for examination, diagnostics and treatment for adiposity.

**Referred by**: General practitioner___ Community healthcare____ .

**Allergies**: no___ yes___ , if yes which: ____________________________________ .

**Previous treatment for overweight or obesity** (which): ________________________________.

**Familial predispositions:**

(Do any of the following family members have or previously had these conditions? Please tick)

|  | **Deceased** | **Obesity** | **Bariatric surgery** | **Hypertension** | **Diabetes Mellitus type 2** | **Stroke** | **Dyslipidaemia** |
| --- | --- | --- | --- | --- | --- | --- | --- |
| **Mother** |  |  |  |  |  |  |  |
| **Father** |  |  |  |  |  |  |  |
| **Grandmother**  **(Mothers side)** |  |  |  |  |  |  |  |
| **Grandfather**  **(Mothers side)** |  |  |  |  |  |  |  |
| **Grandmother**  **(Fathers side)** |  |  |  |  |  |  |  |
| **Grandfather**  **(Fathers side)** |  |  |  |  |  |  |  |
| **Mother’s sister** |  |  |  |  |  |  |  |
| **Mother’s brother** |  |  |  |  |  |  |  |
| **Father’s sister** |  |  |  |  |  |  |  |
| **Father’s brother** |  |  |  |  |  |  |  |
| **Sibling** |  |  |  |  |  |  |  |
| **Sibling** |  |  |  |  |  |  |  |

(Adopted no___ yes___. Do not ask directly, but if it comes up during the dispositions or other interviews, check here)

**Mother**: height: ___________cm. Social security number:____________

Weight (self-reported):____________ kg Weight (measured) :____________ kg

Occupation: ____________

**Father:** height: ___________cm. Social security number:____________

Weight (self-reported):____________ kg Weight (measured):____________ kg

Occupation: ____________

**Ethnicity:** Native yes no if no, which ethnicity____________

Is/was your mother native yes no if no, which ethnicity ____________

Is/was your father native yes no if no, which ethnicity ____________

Is/was your mother’s mother native yes no if no, which ethnicity ____________

Is/was your mother’s father native yes no if no, which ethnicity ____________

Is/was your father’s mother native yes no if no, which ethnicity ____________

Is/was your father’s father native yes no if no, which ethnicity ____________

**Pregnancy:** GA: ____ +____ *(weeks+days).* Birth weight: _________ g. Birth length: _______ cm.

**Pregnancy complications**: ___preeclampsia, ____other:____________________

Mother: ____DM1, ____DM2, ____GDM, ____Other types of diabetes:

Birth: ___ uncomplicated, ___ emergency caesarean, ___ elective caesarean_____, ventouse delivery___ .

**Breastfeeding**: ____________months.

**Child vaccination program completed**: yes_____ no____ partially_____ .

**Previous or current long-term illnesses:**

_______________________________________________________________________________ .

**Debut of the child’s overweight**: age: _______ years.

**Height and weight during the first 5 years of life**:

| **Age** | **3 months** | **5 months** | **1 years** | **2 years** | **3 years** | **4 years** | **5 years** |
| --- | --- | --- | --- | --- | --- | --- | --- |
| **Date** |  |  |  |  |  |  |  |
| **Height** |  |  |  |  |  |  |  |
| **Weight** |  |  |  |  |  |  |  |

Height and weight, preferably 5 years retrospectively

Date Age Height Weight

Date Age Height Weight

Date Age Height Weight

Date Age Height Weight

Date Age Height Weight

**Daily diet**

Pickiness (Is the child perceived as picky?): yes______ no_______

Breakfast: (days per week: __ ) Content:

10 am snack/meal: (days per week: __ ) Content:

Lunch: (days per week: __ ) Content:

Afternoon snack/meal: (days per week: __ ) Content:

Dinner: (days per week: __ ) Content:

Before bedtime: (days per week: __ ) Content:

Vegetables/Salad (for dinner): days per week: __

Portion size and number of servings (dinner): (fill in the number)

Small: ______ medium: ______ large: _____

**Eating behaviours:** Comfort eating: yes___ no___. Overeating: yes___ no___ .

Skipping meals: yes__ no___ . Rapid eating: yes___ no___ .

**Fast food** (pizza, burger, kebab, French fries, etc.): ______ times per month.

**Snacking** (cake, ice cream, popcorn, crisps, chocolate): _______ times per week. **Candy**: _______ times per week.

**Fruit**: _______ pieces per day.

**Drinks** **Sugar sweetened**:

Soda: ___ *times/week, total ___ liter/week.*

Juice: ___ *times/week, total ___ liter/week.*

Iced tea: ___ *times/week, total ___ liter/week.*

Lemonade: ___ *times/week, total ___ liter/week.*

Cocoa: ___ *times/week, total ___ liter/week.*

**Diet products**:

Soda: ____ *times/week, total ____ liter/week.*

Juice: ____ *times/week, total ____ liter/week.*

Iced tea: ____ *times/week, total ____ liter/week.*

Lemonade: ____ *times/week, total ____ liter/week.*

Cocoa: ____ *times/week, total ____ liter/week.*

Milk (*liter/week)*: Skimmed: ____ Mini: ____ Low fat: ____ Whole: ___.

**Physical activity**

**Organised sport:** __________________________________

(Examples: aerobics, badminton, basketball, boxing, cycling, dancing, fitness, football, gymnastics, handball, ice hockey, karate, running, riding, swimming, tennis, volleyball).

Intensity level: low____ *hours/week*, medium ____*hours/week*, high___ *hours/week.*

**Other physical activities** (trampoline, running, dancing etc.): ____ *hours/week.*

**Transportation:**

Bikes to/from school: yes____ no___. Total amount km/day (on average): _______ .

Walks to/from school: yes____ no___. Total amount km/day (on average): ______ .

Driven to school: yes___ no____ .

Dangerous road to school: yes___ no____, distance to school:______ km.

Bicycling (besides to/from school): amount of km/day (on average): ______ .

Walking (besides to/from school): amount of km/day (on average): _______ .

TV/PC/tablet: time in front of a screen: _______ hours/day.

Amount of hours sleep ___________ hours/night.

**Medication** no___ yes___, if yes, which: ___________________________________________.

**Alcohol** no___ yes___, if yes: number of units per week:______.

**Smoking habits**

Does the child/adolescent smoke? no___ yes___ amount per week:____

If no: has the child/adolescent regularly smoked previously? no__ yes__

**Passive** **smoking**

Does the father smoke: no____ yes____ amount per week: ________.

Does the mother smoke: no____ yes____ amount per week: ________ .

Are there anybody else at home who smokes (for example siblings): no____yes____ amount per week: ________ .

Did the mother smoke during the pregnancy: no____ yes____.

**Family structure and school attendance:**

Number of siblings (full, biological): older____/younger____.

Number of half-siblings: older____/younger____. Number of stepsiblings: older___/younger____.

Lives with: mother and father____ mother____, father____, shared_____, alone ____, grandparents____, foster family _____, or other_____

Parental status: Lives together____, married____, divorced____, separated____, or single____

Custody: Shared _____, mothers_____ , or fathers_____ .

If the parents are separated/non-cohabiting, how is the cooperation?

Good_____ or problematic_____.

Is there any psychiatric disease in the family: Mother: ________. Father: ________.

Has a close relative of the child passed away recently? yes___ no___.

Does the child/adolescent currently attend school: yes___ no___.

Grade: _______. Special class: yes___ no___.

Change of school: yes___ no___. If yes, how many times: _____

and what years? ____________________ .

Moving: yes___ no___ If yes, how many times____ and what years? ___________.

**Bullying:** Are you being bullied: yes____ no____. Are you being excluded: yes____ no____ . Have you become used to bullying: yes___ no___ . Are you lonely: yes___ no___.

**Objective measures:**

Weight (kg): ___________Height (cm): ___________.

Waist circumference (cm): ___________ Hip circumference (cm): ___________.

**Treatment plan:**

1.

2.

3.

4.

5.

6.

7.

8.

9.

10.

11.

12.

13.

14.

15.

16.

17.

18.

19.

20.
