# Supplementary material for: A hospital-based child and adolescent overweight and obesity treatment protocol transferred into a community healthcare setting
Source: PLoS One. 2017 Mar 6;12(3):e0173033. doi: 10.1371/journal.pone.0173033 (PMC5338817; doi:10.1371/journal.pone.0173033)
Supplement: S2 File — (DOCX) [file pone.0173033.s002.docx]

**Study protocol**

**Title**

**Transferring a Hospital-based Child and Adolescent Overweight and Obesity Treatment into a Community-based Treatment: A Study Protocol.**

**Authors**

Pernille M Mollerup, Cæcilie Trier, Tenna RH Nielsen, Christine Bøjsøe, Jennifer L Baker, and Jens-Christian Holm.

**Affiliations**

^1^ The Children’s Obesity Clinic, Department of Paediatrics, Copenhagen University Hospital Holbæk, Smedelundsgade 60, DK-4300 Holbæk, Denmark

^2^ Novo Nordisk Foundation Center for Basic Metabolic Research, Section of Metabolic Genetics, University of Copenhagen, Universitetsparken 1, DK-2200 Copenhagen, Denmark

^3^ Institute of Preventive Medicine, Bispebjerg and Frederiksberg Hospitals, The Capital Region, Nordre Fasanvej 57, DK-2000 Frederiksberg, Denmark

^4^ Faculty of Health and Medical Sciences, University of Copenhagen, Blegdamsvej 3B, DK-2200 Copenhagen, Denmark

**Correspondence to** Pernille M Mollerup

Telephone-number: 0045 20 91 03 98. Email: [Pmm@regionsjaelland.dk](mailto:Pmm@regionsjaelland.dk)

Address: The Children’s Obesity Clinic, Department of Paediatrics, Copenhagen University Hospital Holbæk, Smedelundsgade 60, DK-4300, Holbæk, Denmark.

**Abbreviations**

BMI – Body mass index

SDS – Standard deviation score

TCOCT – The Children’s Obesity Clinic Treatment

**Background**

The prevalence of children and adolescence with overweight and obesity has reached alarmingly high levels worldwide, and this has led to an urgent need for improvements and availability and accessibility in both prevention and treatment [1]. Child and adolescent overweight and obesity are accompanied by co-morbidities such as dyslipidaemia [2], fatty liver disease [4,5], hypertension [5], and impaired glucose tolerance [6], increasing the risk of cardiovascular disease, diabetes, and premature death in adulthood [7,8]. Furthermore, children and adolescents with overweight and obesity are frequently burdened by severe psychosocial problems and stigmatization [9]. Due to this wide range of complications, treatment of child and adolescent overweight and obesity is a complicated task.

Based upon systematic reviews provided by The Cochrane Collaboration [10] and the American Academy of Pediatrics [11–13], the recommended approach for treating childhood obesity is a family-based behaviour-changing lifestyle intervention. However, results from previous interventions are inconsistent, and often negatively affected by high dropout rates [10]. At The Children’s Obesity Clinic, Copenhagen University Hospital Holbæk, Denmark, a thorough treatment approach, The Children’s Obesity Clinic’s Treatment (TCOCT) protocol, based upon the aforementioned recommendations has been successfully implemented [14]. Since 2008, more than 2,100 overweight and obese children (body mass index (BMI) standard deviation score (SDS) above the 90^th^ percentile) aged 0−24 years have been included in the treatment program, regardless of additional diseases such as asthma, attention deficit disorders, epilepsy, various syndromes, or other complex chronic diseases, which may complicate overweight and obesity treatment [15,16]. Thus, no other eligibility criteria are applied prior to inclusion into treatment. During treatment, the families visit the clinic on average every six weeks. However, the frequency of the visits varies depending on the individual needs of the families [14]. The treatment approach is described in detail elsewhere [14] where it was documented, that after two years of treatment 63% of the children had reduced their BMI SDS by 0.40 in boys and 0.25 in girls, regardless of their baseline BMI SDS, and socioeconomic status [14]. Equally essential, medical co-morbidities such as dyslipidaemia [2], the amount of fat in the liver and visceral adipose tissue [17], and hypertension [18] were reduced during treatment.

Due to considerations of accessibility and capacity, recent recommendations have addressed the significance of the community level of healthcare services in the identification and treatment of child and adolescent overweight and obesity [19–21]. However, evaluations of community-based treatment programs are sparse, and many are limited by lack of adequate power, incomplete documentation, or a short follow-up period [22]. In the United Kingdom, 2010, a nine-week intervention (“Mind, Exercise, Nutrition…. Do it (MEND)”), consisting of 18 two-hour group sessions of education and physical activity followed by 12 weeks of free access to a local community swimming pool, in 116 children aged 8−12 years with obesity (BMI above the 98^th^ percentile) was evaluated [23]. The study demonstrated that children in the intervention group reduced their BMI SDS by a mean of 0.30, but at the 12-month follow-up the mean reduction had decreased to 0.23. In Australia, 2011, another program (“Loozit”) was evaluated in 151 adolescents aged 13−16 years with overweight or moderate obesity (BMI SDS: 1.0−2.5) [24,25]. This program included seven 75-minutes group sessions held weekly followed by sessions every three months with or without additional telephone contacts, texts messages, or emails. After two months, BMI SDS was reduced by 0.05 [24], after one year by 0.09 [25]. Hence, the feasibility of effective community-based overweight and obesity treatment has been questioned [26–28].

Furthermore intensive treatment programs are often more effective, but may not be feasible outside hospital or research settings [28]. During the TCOCT program, each family spends an average of five to six hours per year on consultations at the clinic [14]. Importantly, this is not due to a less extensive treatment approach, but rather to a very individualised program, including specific recommendations about the implementation of the lifestyle changes into the everyday lives of the families. As example, no physical activity sessions are held, but instead specific agreements are made on how the families will introduce the physical activities themselves by using their local sports facilities. Hence, time invested in consultation hours during the TCOCT program are few [14]. Therefore, this treatment program may provide a viable and feasible treatment modality for other healthcare settings. However, whether the TCOCT is effective, when it is applied in the community healthcare setting, has yet to be established.

The feasibility of transferring an effective hospital-based intervention into a community healthcare setting has been evaluated in a pilot-study by Banks *et al.* in 2012 [27]. In this study, the community-based intervention was delivered by dietitians, nurses, and physical trainers who were thoroughly trained. The one-year intervention consisted of five consultations, during which the families consulted all three health care practitioners in turn. Seventy-six children and adolescents with obesity (BMI above the 98^th^ percentile) aged 5−16 years were included. In the group treated at the hospital, BMI SDS was reduced by a mean of 0.15, and in the group treated at the community healthcare centre, BMI SDS was reduced be a mean of 0.17. Although the reductions in BMI SDS were both non-significant which was likely due to a lack of statistical power, the similarity of the results suggests that transferring a hospital-based intervention into a community healthcare setting may be feasible.

**Aim and perspective**

The aim of the present study is to evaluate the effect of the TCOCT protocol applied in a community healthcare setting delivered by community nurses and dieticians. The potential implication is the development of an effective community-based child and adolescent overweight and obesity treatment program.

**Methods**

*Design*

In the present single-armed, prospective study, we will investigate the changes in BMI SDS and waist circumference in more than 800 children and adolescents with overweight and obese during 1.5 years of community-based overweight and obesity treatment based upon the TCOCT protocol.

*Ethics*

The study will be conducted as a part of the research activities in the research project, *The Danish Childhood Obesity Biobank*, which is approved by the regional Danish Ethics Committee (Protocol ID SJ-104; Suppl. 1). The research activities in the present study are approved in a supplementary protocol for *The Danish Childhood Obesity Biobank* (Suppl. 2), and are approved by the Danish Data Protection Agency. Both the present study and *The Danish Childhood Obesity Biobank* study are registered at Clinicaltrials.gov (ID number NCT02013843 and NCT00928473, respectively).

*Establishment of a community-based overweight and obesity treatment*

In Denmark, the community level of healthcare is delivered at municipal healthcare centres. This level of healthcare provides a variety of healthcare services and social services including services to children and adolescents with special needs. Nonetheless, to date, no standard community-based child and adolescent overweight and obesity treatment program exist.

This study was initiated by The Children’s Obesity Clinic and the Region Zealand. Hence, the healthcare managements in the Region Zealand were invited to participate in the project and thus, the participating healthcare centres were not randomly selected. Participation of the healthcare centres was voluntary and four healthcare centres agreed to participate. Subsequently, four municipal healthcare centre’s managements from outside the Region Zealand contacted The Children’s Obesity Clinic at their own initiative to establish an overweight and obesity treatment program based upon the TCOCT protocol, and the healthcare centres in these municipalities were included in the study. Participation in the study required the healthcare centre to finance the necessary equipment, and the working hours needed for the training and supervision of the nurses and dieticians, for the treatment of approximately 100 children and adolescents with overweight and obesity during the study period. It was estimated that approximately two weeks were needed for the training and supervision of the nurses and dietitians (fig 1), and approximately six hours should to be allocated for the treatment of each child/adolescent per year. No other eligibility criteria for the healthcare centres to engage into the study were applied. The establishing of the community-based overweight and obesity treatment program at the healthcare centres started January 2012 and will end January 2013.

*Training and supervision of the community personnel*

The training of the community personnel will consist of theoretical and practical courses (figure 1). Afterwards, an experienced nurse from The Children’s Obesity Clinic will supervise selected consultations at the healthcare centres throughout the study period to ensure the correct application of the treatment protocol and to further expand the skills of the nurses and dieticians.

**Figure 1**. The training of the community personnel

*Patients*

From June 2012 until January 2015, approximately one hundred children and adolescents with overweight and obesity will be included at each of the eight healthcare centres and treated throughout the study period or until dropout. The criteria for engaging into treatment is an age from 3-18 years and a BMI equal to or above the 85^th^ percentile for age and sex according to Danish reference charts [29]. There are no other eligibility criteria*.* The treatment continues until the child or adolescents achieves a stable normal weight (arbitrary defined as a BMI below the 85^th^ percentile for sex and age [30]), until the child/family wish to end treatment, repeatedly neglect appointments, or until the adolescent turns 19 years.

*Referral of patients*

The children and adolescents will be referred to the treatment by general practitioners, school nurses, or directly from the families to their local healthcare centre. An information-brochure about the offer of overweight and obesity treatment in each municipality will be available on the national website for community healthcare services and as leaflets at the healthcare centres [31].

*The community-based intervention*

The treatment program will be based upon the TCOCT protocol [14]. At the first visit the will families consult with a nurse for 1-1½ hours. At this visit, the nurse completes a comprehensive questionnaire in collaboration with the family during a structured interview. The aim of this interview is to identify all lifestyle changes necessary to optimise the child or adolescent’s life to achieve weight loss. Based upon this, the nurse establishes an individually tailored treatment plan for the family, containing 15-20 items of advice addressing the necessary lifestyle changes. Examples of lifestyle changes are changes allowances, dietary habits, lunch bags, physical activities, screen time and other sedentary behaviours, sleep time, snacking, social activities, and means of transportation. Approximately six weeks later, the families consult with a dietitian for one hour. After this, the frequency of ½ hour consultations will be individualized i.e. will depend on the current needs and challenges in each family. At all consultations, the treatment plan will be evaluated and adjusted. Annually (+/- approximately two months), all families consult with a nurse for ½-1 hour. At this consultation another comprehensive questionnaire based interview will be conducted to evaluate how the lifestyle changes are implemented in daily life.

*Measurements and documentation*

At all consultations, height will be measured to the nearest 0.1 cm on a Tanita^®^ HR100 (Tanita^®^) stadiometer and weight will be measured to the nearest 0.1 kilogram on a Tanita^®^ BC418 scale (Tanita^®^). Hip circumference will be measured at the widest part of the hips using a non-elastic measuring tape. Waist circumference will be measured at the level of the umbilicus after a light exhalation in the standing position. All measurements will be performed wearing light indoor clothes with empty pockets and without shoes.

*Statistics*

BMI will be calculated as weight in kilograms divided by height in metres squared. Based ipon Danish reference BMI charts [29], the BMI SDS will be calculated by the LMS method, which converts the skewed BMI distribution into a normal distribution by sex and age using the median, the coefficient of variation, and a measure of the skewness provided by a Box Cox Power Plot. Baseline characteristics and measurements will be compared by chi-squared analyses for categorical data, and by relevant parametric and non-parametric tests for continuous data. Logarithmic transformation(s) will be used when appropriate. The changes in BMI SDS, and waist circumference will be analysed in linear mixed models. The potential confounders of the child’s age, pubertal stage, sex, and socioeconomic status will be included in the models when appropriate.

*Sample size*

Based on a power calculation with the minimal relevant difference from baseline to follow-up, in BMI SDS, arbitrary defined as 0.10 BMI SDS, and an SD of 0.45 (derived from previous evaluations at The Children’s Obesity Clinic), a sample size of 215 patients is needed. Assuming a dropout of 30% after one year, a sample size of 307 patients is needed. Due to the non-parallel reductions in BMI SDS in boys and girls observed at The Children’s Obesity Clinic [14], sex-stratified analyses are prioritised and a larger sample size will enable sub-studies. In adults, controversies exist regarding the health benefits and harms of weight reductions in patients with overweight and obesity without apparent complications [32]. In children, comprehensive meta-analyses concluded that behaviour changing lifestyle interventions, such as the TCOCT protocol, are unlikely to cause any harm [26,28]. Further, overweight and obesity treatment may reduce cardiovascular risk factors [2,18,33] and may improve the quality of life [34–36]. Due to these considerations we found it ethically acceptable to include a larger number of patients. Further, due to considerations regarding the personnel’s experience in treating overweight and obesity in accordance with the TCOCT protocol, we aimed at including approximately one hundred children and adolescents at each healthcare centre.

*Date collection*

All data will be continuously collected at the healthcare centres and entered into a pre-existing database at The Children’s Obesity Clinic.

*Funding*

The study is partially funded by the Region of Zealand (suppl. 3) and by the Region of Zealand Health Scientific Research foundations. The community healthcare centres are responsible for allocating the necessary equipment, and the working hours needed for the training and supervision of the nurses and dieticians, for the treatment of approximately 100 children and adolescents with overweight and obesity during the study period.

*Time schedule of the study*

January 2012 to January 2013:

Establishing a community-based treatment for children and adolescents with overweight and obesity based upon the TCOCT protocol at healthcare centres in eight municipalities in Denmark.

July 2012 to January 2015:

Enrolment of children and adolescents into community-based overweight and obesity treatment. Data are continuously collected from the healthcare centres to The Children’s Obesity Clinic.

March 2015:

End of follow-up on the enrolled children.

(The treatment program is ongoing)

From march 2015:

Final data collection and data analyses.

**Supplemental:**

**Suppl. 1:**

English translation of the approval of *The Childhood Obesity Biobank* by the Ethics Committee.

Review of protocol no. SJ-104.

**Suppl. 2:**

English translation of the approval of the activities in the present study as supplement to the approval *The Childhood Obesity Biobank* by the Ethics Committee (supplemental no. 10).

**Suppl. 3:**

English translation of the agreement of collaboration between The Children’s Obesity Clinic and the Region Zealand.

**References**

1. Rokholm B, Baker J, Sørensen T. The levelling off of the obesity epidemic since the year 1999–a review of evidence and perspectives. Obes Rev. 2010;11: 835–846. doi:10.1111/j.1467-789X.2010.00810.x

2. Nielsen TRH, Gamborg M, Fonvig CE, Kloppenborg J, Hvidt KN, Ibsen H, et al. Changes in lipidemia during chronic care treatment of childhood obesity. Child Obes. 2012;8: 533–41. doi:10.1089/chi.2011.0098

3. Schwimmer JB, Deutsch R, Kahen T, Lavine JE, Stanley C, Behling C. Prevalence of fatty liver in children and adolescents. Pediatrics. 2006;118: 1388–93. doi:10.1542/peds.2006-1212

4. Bille DS, Chabanova E, Gamborg M, Fonvig CE, Nielsen TRH, Thisted E, et al. Liver fat content investigated by magnetic resonance spectroscopy in obese children and youths included in multidisciplinary treatment. Clin Obes. 2012;2: 41–49. doi:10.1111/j.1758-8111.2012.00038.x

5. Babinska K, Kovacs L, Janko V, Dallos T, Feber J. Association between obesity and the severity of ambulatory hypertension in children and adolescents. J Am Soc Hypertens. 2012;6: 356–63. doi:10.1016/j.jash.2012.08.002

6. Caprio S, Bronson M, Sherwin RS, Rife F, Tamborlane W V. Co-existence of severe insulin resistance and hyperinsulinaemia in pre-adolescent obese children. Diabetologia. 1996;39: 1489–97. Available: http://www.ncbi.nlm.nih.gov/pubmed/8960831

7. Baker JL, Olsen LW, Sørensen TIAA, Institute of Preventive Medicine, Center for health and Society C. Childhood Body-Mass Index and the Risk of Coronary Heart Disease in Adulthood. N Engl J Med. 2007;357: 2329–2337. doi:10.1056/NEJMoa072515.Childhood

8. Franks P, Hanson R. Childhood Obesity, Other Cardiovascular Risk Factors, and Premature Death. N Engl J Med. 2010;362: 485–493. doi:10.1056/NEJMoa0904130.Childhood

9. Grønbæk HN, Holm J-C. [Psychological consequences of severe overweight in teenagers]. Ugeskr Laeger. 2011;173: 1785–1791. Available: http://www.ncbi.nlm.nih.gov/pubmed/21689505

10. Oude Luttikhuis H, Baur L, Jansen H, Shrewsbury VA, O’Malley C, Stolk RP, et al. Interventions for treating obesity in children. Cochrane Libr. 2009; CD001872. doi:10.1002/14651858.CD001872.pub2

11. Davis MM, Gance-Cleveland B, Hassink S, Johnson R, Paradis G, Resnicow K. Recommendations for prevention of childhood obesity. Pediatrics. 2007;120 Suppl: S229-53. doi:10.1542/peds.2007-2329E

12. Spear BA, Barlow SE, Ervin C, Ludwig DS, Saelens BE, Schetzina KE, et al. Recommendations for Treatment of Child and Adolescent Overweight and Obesity. Pediatrics. 2007;120: S254–S288. doi:10.1542/peds.2007-2329F

13. Krebs NF, Himes JH, Jacobson D, Nicklas T a, Guilday P, Styne D. Assessment of child and adolescent overweight and obesity. Pediatrics. 2007;120 Suppl: S193-228. doi:10.1542/peds.2007-2329D

14. Holm J-C, Gamborg M, Bille DS, Grønbæk HN, Ward LC, Faerk J. Chronic care treatment of obese children and adolescents. Int J Pediatr Obes. 2011;6: 188–196. doi:10.3109/17477166.2011.575157

15. Reinehr T, Brylak K, Alexy U, Kersting M, Andler W. Predictors to success in outpatient training in obese children and adolescents. Int J Obes Relat Metab Disord. 2003;27: 1087–92. doi:10.1038/sj.ijo.0802368

16. Pernilla D, Jan K, Ekblom Ö, Marcus C, Danielsson P, Kowalski J, et al. Response of severely obese children and adolescents to behavioral treatment. Arch Pediatr Adolesc Med. 2012;166: 1103–8. doi:10.1001/2013.jamapediatrics.319

17. Bille DS. Aspects of human obesity, PhD thesis. University of Copenhagen, Faculty of Health and Medical Sciences. 2012.

18. Hvidt KN, Olsen MH, Ibsen H, Holm J-C. Effect of changes in BMI and waist circumference on ambulatory blood pressure in obese children and adolescents. J Hypertens. 2014;32: 1470–7. doi:10.1097/HJH.0000000000000188

19. Vine M, Hargreaves MB, Briefel RR, Orfield C. Expanding the role of primary care in the prevention and treatment of childhood obesity: a review of clinic- and community-based recommendations and interventions. J Obes. 2013;2013: 172035. doi:10.1155/2013/172035

20. Baker JL, Farpour-Lambert NJ, Nowicka P, Pietrobelli A, Weiss R. Evaluation of the overweight/obese child--practical tips for the primary health care provider: recommendations from the Childhood Obesity Task Force of the European Association for the Study of Obesity. Obes Facts. 2010;3: 131–7. doi:10.1159/000295112

21. Glickman D, Parker L, Sim LJ, Del H, Cook V. Accelerating Progress in Obesity Prevention : Solving the Weight of the Nation. 2012.

22. Katzmarzyk PT, Barlow S, Bouchard C, Catalano PM, Hsia DS, Inge TH, et al. An evolving scientific basis for the prevention and treatment of pediatric obesity. Int J Obes (Lond). 2014;38: 887–905. doi:10.1038/ijo.2014.49

23. Sacher PM, Kolotourou M, Chadwick PM, Cole TJ, Lawson MS, Lucas A, et al. Randomized controlled trial of the MEND program: a family-based community intervention for childhood obesity. Obesity. 2010;18 supplem: S62-8. doi:10.1038/oby.2009.433

24. Shrewsbury VA, Nguyen B, O’Connor J, Steinbeck KS, Lee A, Hill AJ, et al. Short-term outcomes of community-based adolescent weight management: The Loozit® Study. BMC Pediatr. 2011;11: 13. doi:10.1186/1471-2431-11-13

25. Nguyen B, Shrewsbury VA, O’Connor J, Steinbeck KS, Lee A, Hill AJ, et al. Twelve-month outcomes of the loozit randomized controlled trial: a community-based healthy lifestyle program for overweight and obese adolescents. Arch Pediatr Adolesc Med. American Medical Association; 2012;166: 170–7. doi:10.1001/archpediatrics.2011.841

26. Flynn M a T, McNeil D a, Maloff B, Mutasingwa D, Wu M, Ford C, et al. Reducing obesity and related chronic disease risk in children and youth: a synthesis of evidence with “best practice”recommendations. Obes Rev. 2006;7 Suppl 1: 7–66. doi:10.1111/j.1467-789X.2006.00242.x

27. Banks J, Sharp D, Hunt L, Shield J. Evaluating the transferability of a hospital-based childhood obesity clinic to primary care: a randomised controlled trial. Br J Gen Pr. 2012; 6–12. doi:10.3399/bjgp12X616319.Conclusion

28. Luttikhuis HO, Baur L, Jansen H, Shrewsbury VA, O’Malley C, Stolk RP, et al. Interventions for treating obesity in children. Cochrane Database Syst Rev. 2009; CD001872. doi:10.1002/14651858.CD001872.pub2

29. Nysom K, Mølgaard C, Michaelsen KF, Hutchings B, Andersen E. [Body mass index. Reference values for 0-45-year-old Danes]. Ugeskr Laeger. 2002;164: 5773–5777. Available: http://www.ncbi.nlm.nih.gov/pubmed/12523217

30. Nysom K, Mølgaard C, Hutchings B, Michaelsen KF. Body mass index of 0 to 45-y-old Danes: reference values and comparison with published European reference values. Int J Obes Relat Metab Disord. 2001;25: 177–84. doi:10.1038/sj.ijo.0801515

31. The Danish Health authority - [www.sundhed.dk].

32. Sørensen T, Pedersen B, Sandbæk A, Overvad K. Skal overvægtige voksne tabe sig? 2013.

33. Fonvig CE, Chabanova E, Ohrt JD, Nielsen LA, Pedersen O, Hansen T, et al. Multidisciplinary care of obese children and adolescents for one year reduces ectopic fat content in liver and skeletal muscle. BMC Pediatr. 2015;15: 196. doi:10.1186/s12887-015-0513-6

34. Pratt KJ, Lazorick S, Lamson AL, Ivanescu A, Collier DN. Quality of life and BMI changes in youth participating in an integrated pediatric obesity treatment program. Health Qual Life Outcomes. Health and Quality of Life Outcomes; 2013;11: 116. doi:10.1186/1477-7525-11-116

35. Bocca G, Kuitert MWB, Sauer PJJ, Stolk RP, Flapper BC, Corpeleijn E. A multidisciplinary intervention programme has positive effects on quality of life in overweight and obese preschool children. Acta Paediatr. 2014;103: 962–7. doi:10.1111/apa.12701

36. Fullerton G, Tyler C, Johnston CA, Vincent JP, Harris GE, Foreyt JP. Quality of life in Mexican-American children following a weight management program. Obesity (Silver Spring). 2007;15: 2553–6. doi:10.1038/oby.2007.306

**Supplemental 1.**

**
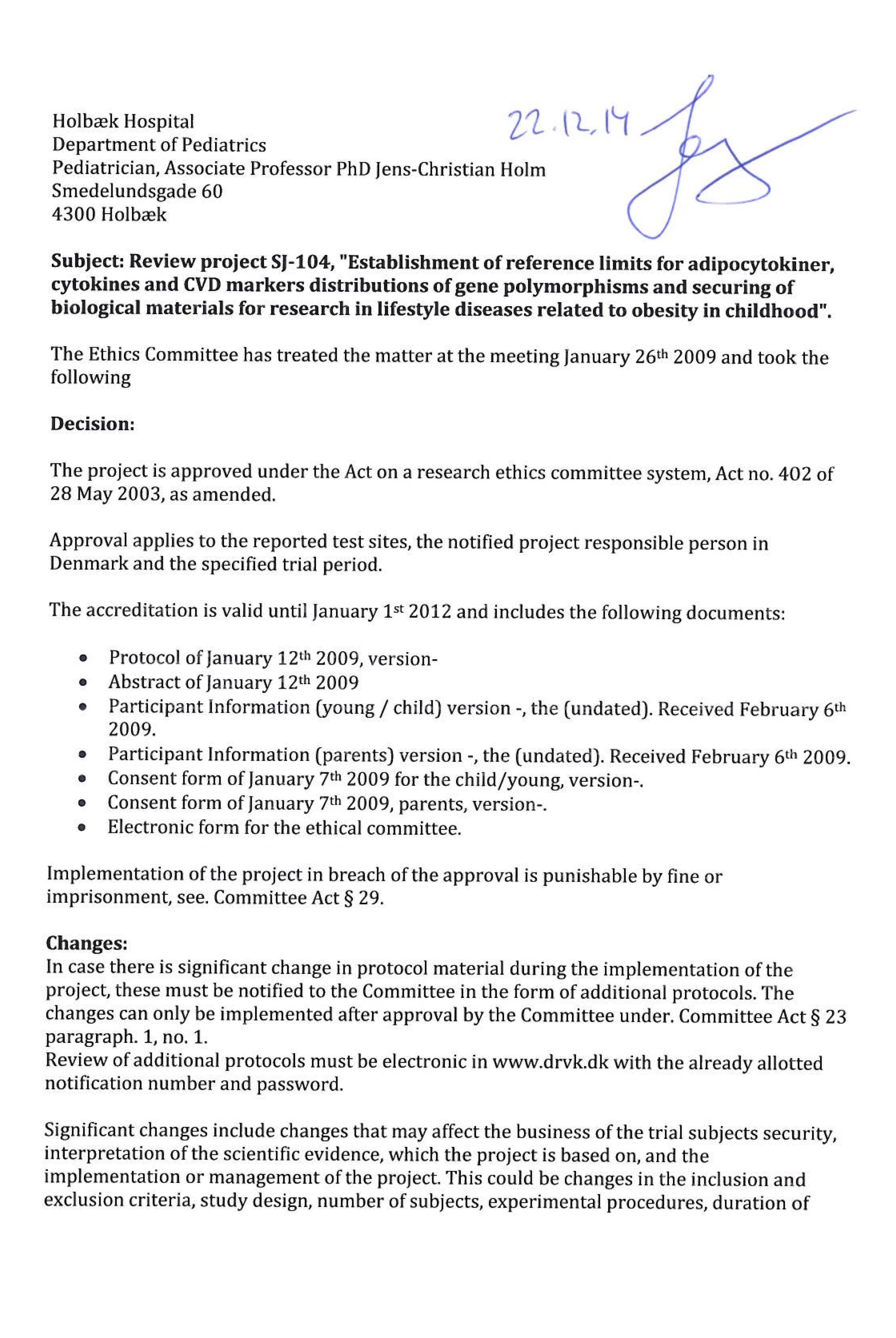
**

**
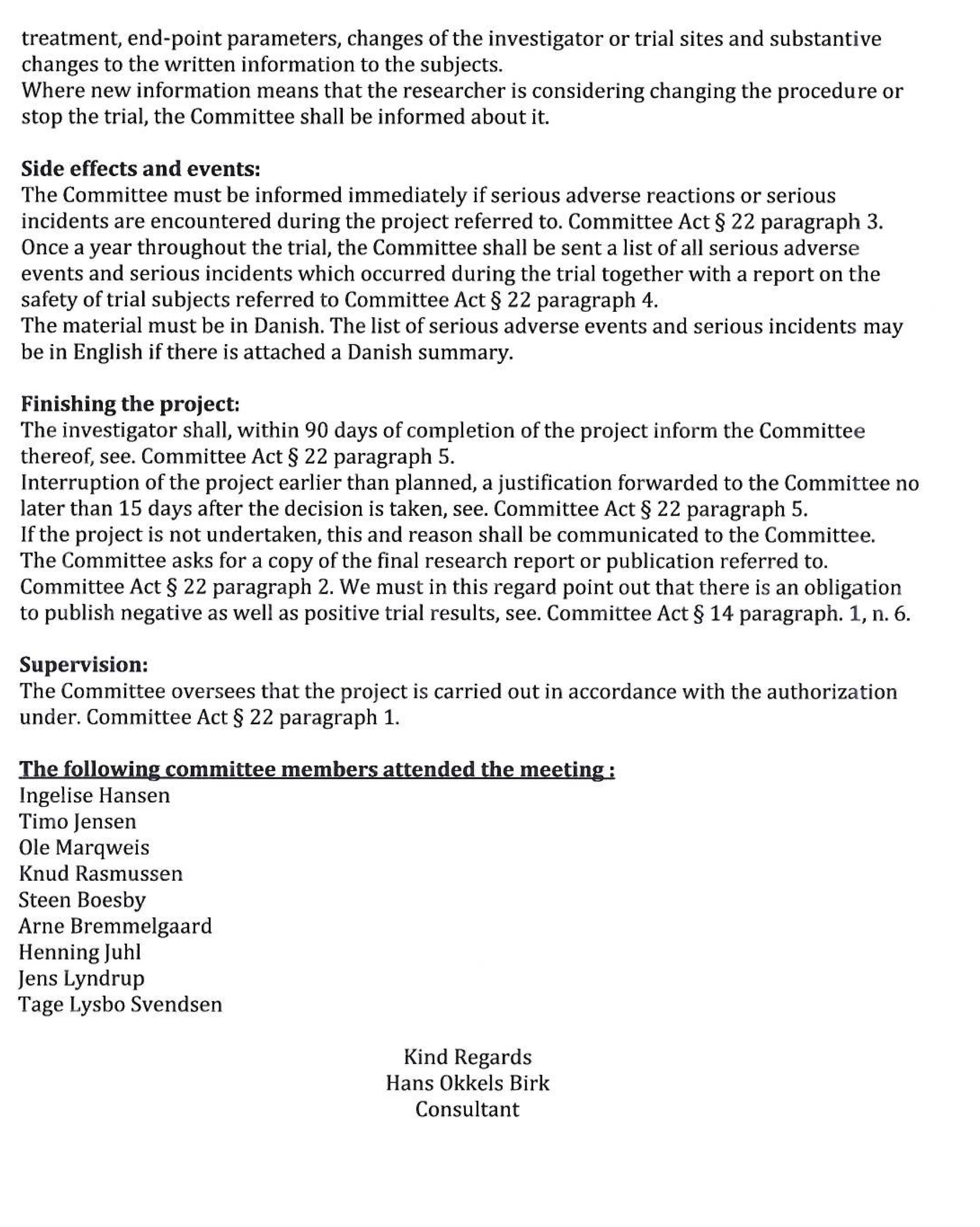
**

**Supplemental 2.**

**
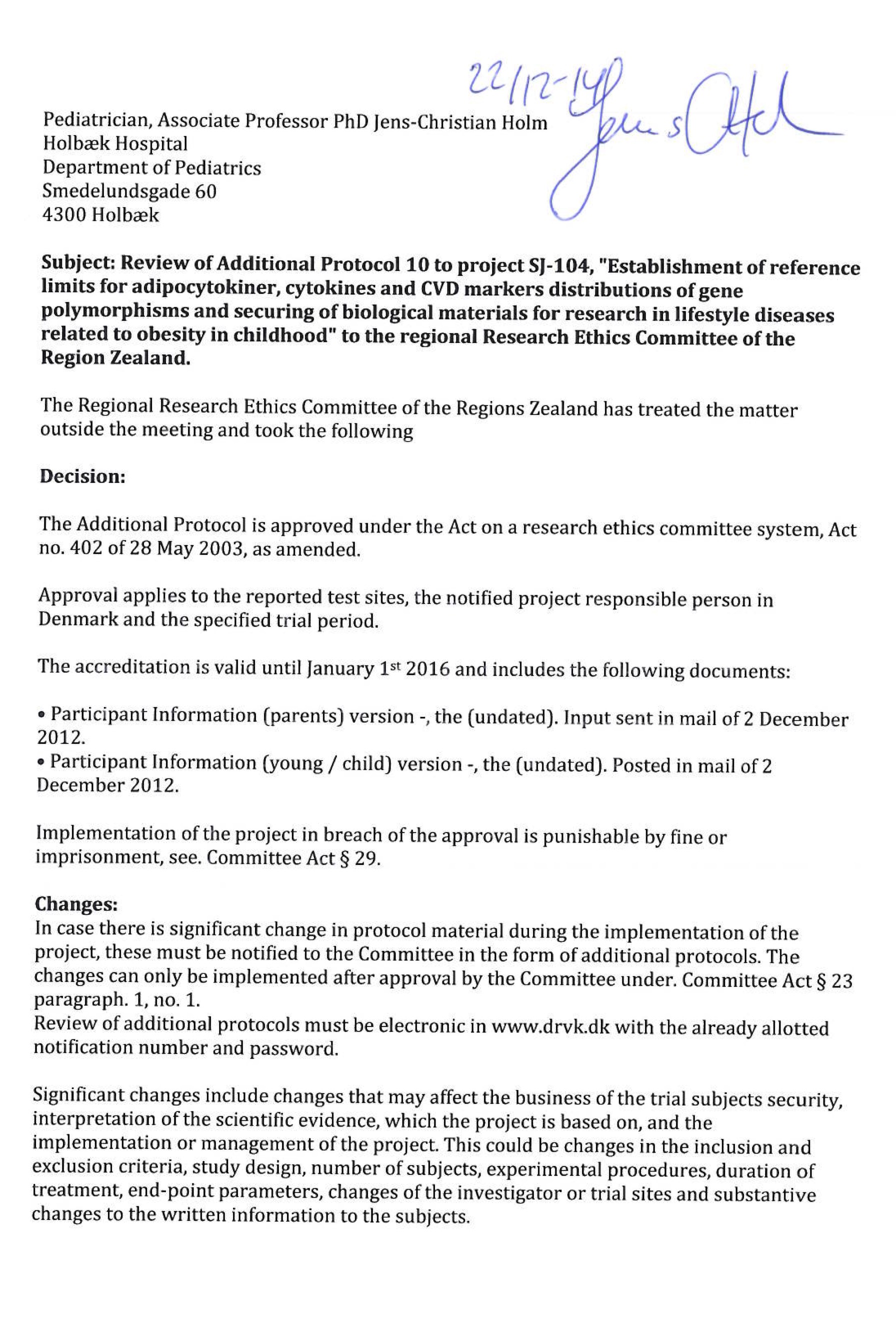
**

**
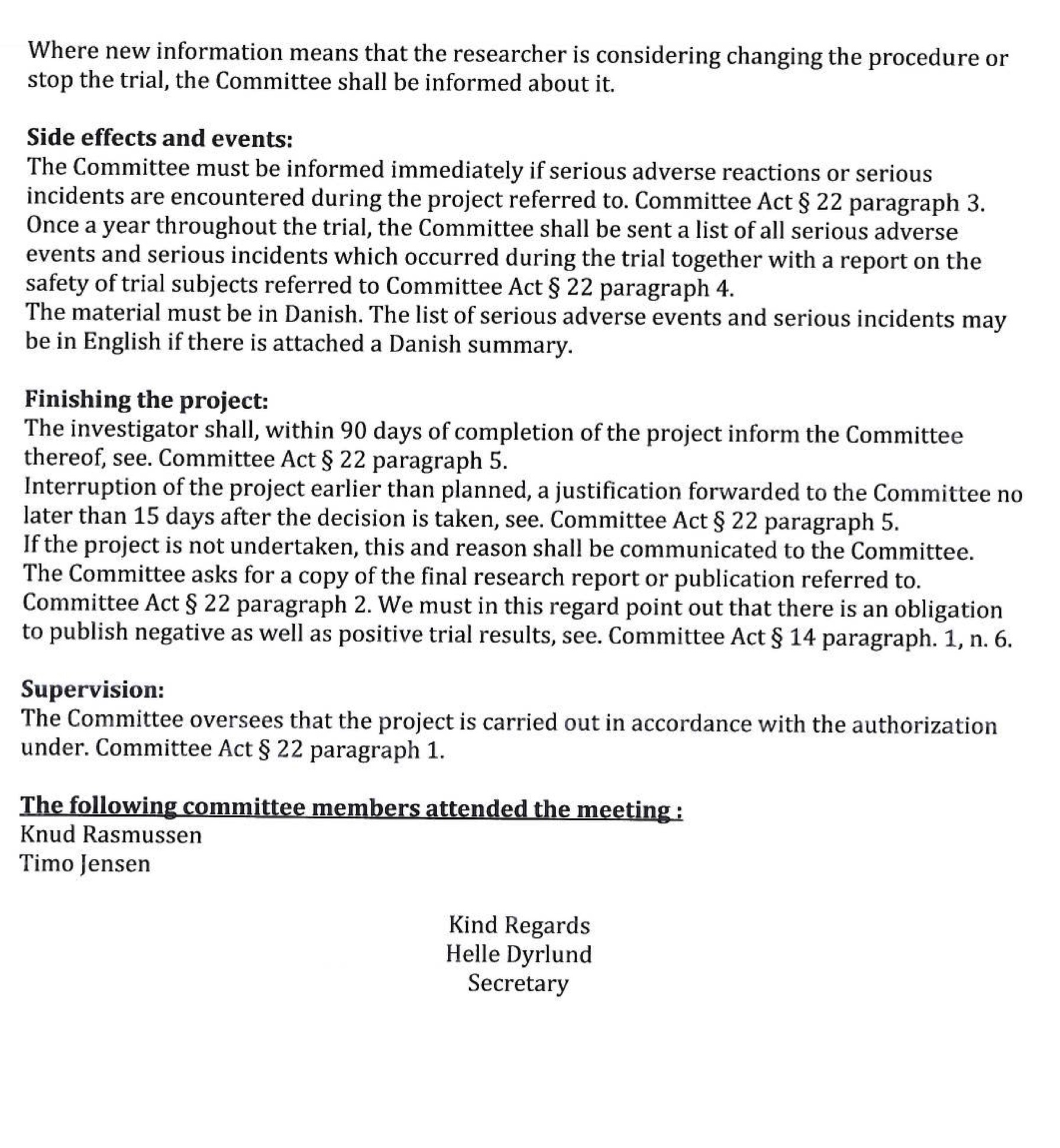
**

**Supplemental 3.**

**
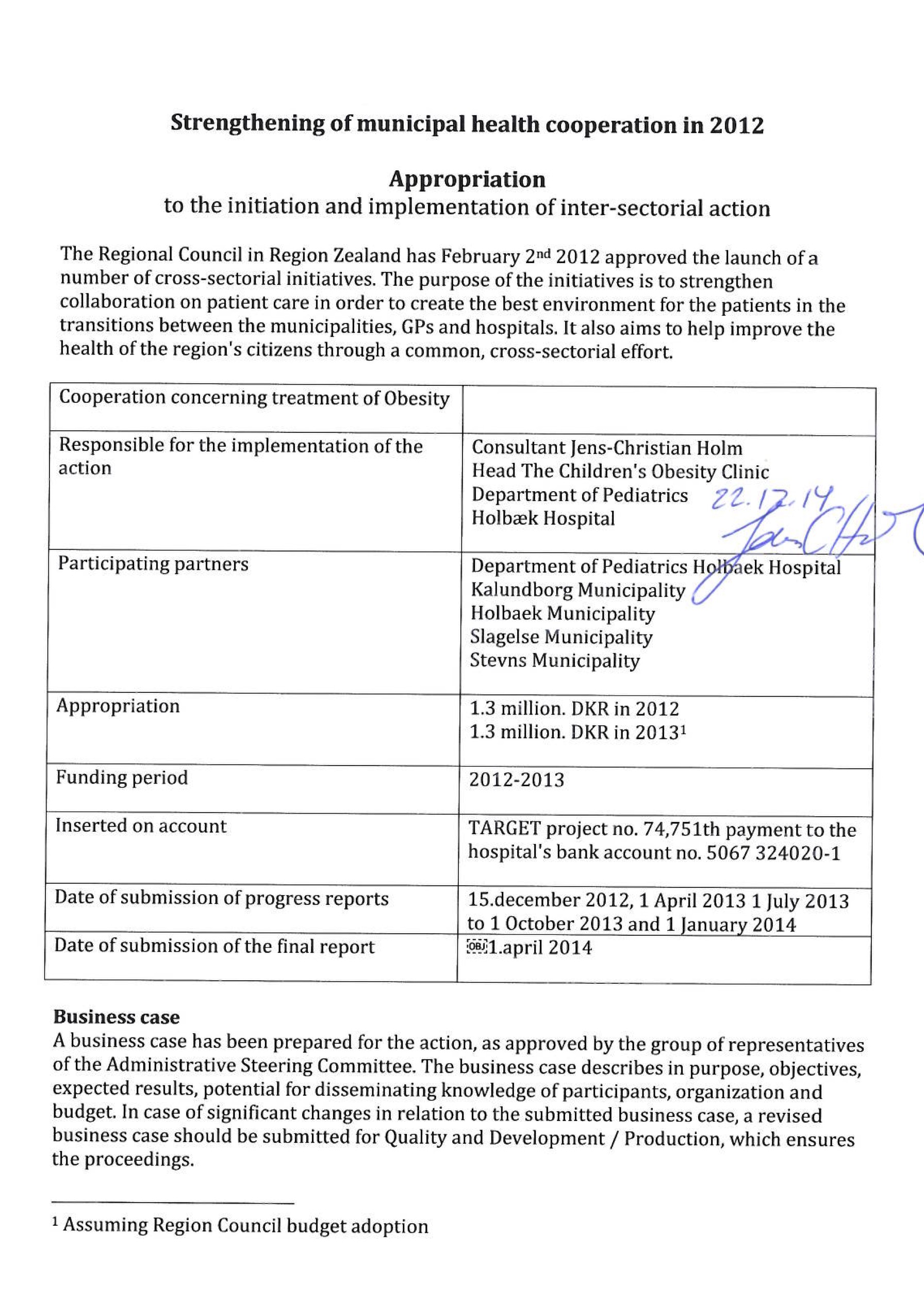
**

**
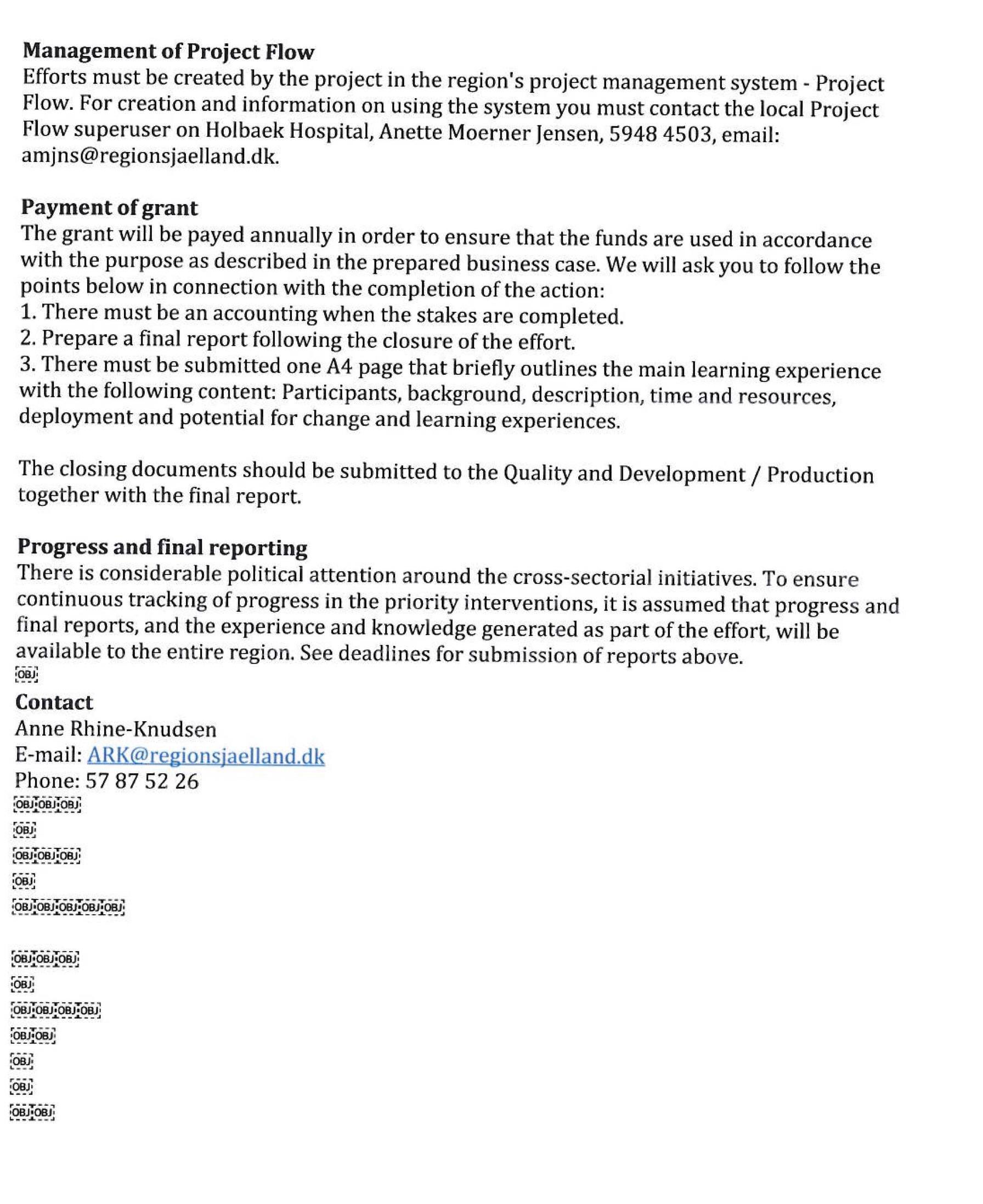
**
