# Supplementary material for: A hospital-based child and adolescent overweight and obesity treatment protocol transferred into a community healthcare setting
Source: PLoS One. 2017 Mar 6;12(3):e0173033. doi: 10.1371/journal.pone.0173033 (PMC5338817; doi:10.1371/journal.pone.0173033)
Supplement: S3 File — (DOCX) [file pone.0173033.s003.docx]

**Research Protocol – Danish Version**

**Behandling af svært overvægtige børn og unge**

**- ”Holbæk-metoden” i kommunalt regi**

**Baggrund**

Udviklingen af overvægt og svær overvægt i barnealderen har de seneste årtier været alarmerende, både i Danmark og globalt [1–4]. Det anslås at der i Danmark er ca. 60.000 svært overvægtige børn og unge, men på trods af dette intet landsdækkende behandlingstilbud. Overvægtige børn og unge plages af svære psykologiske komplikationer, såsom dårlig psykosocial trivsel, social isolation, ensomhed og depression. Livskvaliteten blandt svært overvægtige børn er fundet lige så ringe som blandt børn i behandling for kræft. De har større sociale problemer, alvorligere følelsesmæssige problemer, flere selvmordsforsøg, en mere negativ forventning til egen skolegang og føler sig som dårligere studerende end normalvægtige jævnaldrende [5]. Dertil skal lægges en række alvorlige fysiske komplikationer.

Man har i Enheden for Overvægtige Børn og Unge på Børneafdelingen i Holbæk konstateret at 50% af de svært overvægtige børn har begyndende forhøjet blodtryk [6], 27% har forhøjede fedtstoffer i blodet [7], 44% har mere end 5% fedt i leveren foreneligt med fedtlever [8,9], 74% har forhøjet mængde fedt i musklerne [8] og 18% har forstadier til sukkersyge (Kloppenborg et al, unpublished). Resultatet heraf er en potentielt øget sygelighed og dødelighed i voksenalderen.

*Behandling af overvægt og svær overvægt i primær og sekundær sektor*

Behandling af overvægt hos børn har traditionelt set været en særdeles vanskelig opgave, blandt andet grundet et stort frafald i behandlingen på op til 83% efter bare et års behandling [10]. Dette er uforeneligt med en socialt og økonomisk ansvarlig behandling. Studier af effekten af intervention i primærsektoren er både i Danmark og udlandet sparsom. Flere studier peger på primærsektoren som det ideelle sted for både opsporing og behandling [11–15]. Dette grundet dens umiddelbare tilgængelighed og i forvejen hyppige og løbende kontakt med familierne. Mindre studier og pilot-projekter tyder også på, at intervention i primærsektoren potentielt kan forbedre BMI samt gavne familiernes sundhedstilstand, men yderligere studier til validering efterlyses [16–19].

*Behandling af overvægtige børn og unge – ”Holbæk-metoden”*

I Enheden for Overvægtige Børn og Unge på Børneafdelingen i Holbæk har man stor succes med at behandle svært overvægtige børn og unge uafhængigt af alder, udgangsvægt, sygehistorie og social klasse. 63 % af børnene taber sig efter 2 år, og kun ca. 22 % dropper ud af behandlingen [20]. ”Holbæk-metoden” til behandling af overvægt hos børn og unge er en multidisciplinær indsats, der omfatter blandt andet adfærdsterapi samt en individuel plan for hele familien, som justeres løbende efter behov. Planens indhold varierer hos de forskellige familier, men indeholder altid konkrete aftaler omkring kost og fysisk aktivitet såvel som inaktivitet. Gensidig respekt og empati er centrale elementer i behandlingen, og det er altafgørende at vinde barnets/den unges samt familiens tillid, således at de møder op til de aftalte kontroller. I gennemsnit ses barnet på børneafdelingen hver 6. uge, og der investeres 5-6 timer i barnet/den unge pr år [20]. Børnene henvises fra primærsektoren og børneafdelinger over hele landet, men kapaciteten i såvel Enheden for Overvægtige Børn og Unge som sekundærsektoren generelt er yderst begrænset sammenholdt med behandlingsbehovet både aktuelt og det forventede behov i fremtiden.

**Formål**

Dette studies formål er at undersøge hvorvidt Børneafdelingens behandlingsprincipper og pædagogik, kan implementeres i primærsektoren med sammen succes. Derved søges at definere et valideret behandlingstilbud, som kan fortsættes, videreudvikles og introduceres på landsplan.

**Studie design**

Studiet er et prospektivt of observationelt studie af effekten af den kommunale behandling vurderet ved ændringerne i body mass index standart deviation score BMI SDS og livvide.

*Etablering af et kommunalt behandlingstilbud*

I otte kommuner etableres et behandlingstilbud baseret på ”Holbæk-metoden”. Hertil oplæres sundhedsplejersker og kostvejledere i anvendelse af ”Holbæk-metoden” til behandling initialt ved følgedage på Børneafdelingen, samt teoretisk undervisning, og dernæst ved regelmæssig lokal supervision samt central undervisning og sparring med behandlere på Børneafdelingen. Det er estimeret at ca. 2 uger skal afsættes til

*Studiepopulation*

Hver af de otte kommuner inkluderer i projektperioden minimum ca. 100 børn og unge og behandler disse i projektperioden. Børnene inkluderes efter følgende kriterier:

*Inklusionskriterier*

- Alder mellem 3 og 18 år
- BMI lig med eller over 85-percentilen for køn og alder

*Eksklusionskriterier*

Der er ingen yderligere eksklusionskriterier.

*Undersøgelser af studiepopulationen*

Ved behandlingsstart og efter 1 års behandling udføres følgende undersøgelser.

1. **Højde og vægt måling** (Både højde- og vægtmålinger udføres i let påklædning og uden sko. Stadiometer: Tanita HR001, Vægt: Tanita BC 418. )
2. **Talje og hoftevidde.** (Dette foretages med barnet stående i opret stilling og måles henholdsvis i niveau med umbilicus efter en let udånding, samt på det bredeste sted om hofterne)
3. **Pubertetsudvikling.** (Vurderes ved at barnet/familien udfylder skema med tegninger af forskellige pubertetstrin.)

Ved de alle øvrige kontakter foretages følgende undersøgelser:

1. **Højde og vægt.**
2. **Hofte- og taljemål.**

Derudover udfyldes, som en integreret del af behandlingen, omfattende skemaer, hvorved blandt andet familiære dispositioner, andre sygdomme og sociale forhold, herunder forældrenes arbejde og uddannelse, afdækkes. Ligeledes klarlægges og registreres omhyggeligt relevante forhold omkring kost, samt inaktivitets og aktivitetsvaner i familien.

**Statistiske analyser og styrkeberegning**

I nedenstående styrkeberegning tages udgangspunkt i resultater (BMI SDS standart deviation) fra Enheden for Overvægtige Børn og Unge. Styrkeberegningen er baseret på målet om en signifikant relevant ændring i BMI SDS under 1 års kommunal behandling. En klinisk relevant forskel er sat til 0,15 BMI SDS, signifikansniveau er valgt til 5% og der stiles med en styrke på 90%. På basis af nedenstående beregning skal minimum 129 børn inkluderes i projektet, mens et større antal vil styrke resultaterne yderligere.

Ændringen i BMI SDS og livvide blandt de kommunalt behandlede børn analyseres i linære mixed models, justeret for kendte konfoundere som alder, køn, grad af overvægt og social klasse.

**Etiske overvejelser**

Ved start i den kommunale behandling tilbydes barnet at deltage i dette forskningsprojekt, hvilket betyder at oplysninger fra kommunen videregives til Enheden for Overvægtige Børn, Børneafdelingen, Holbæk. Det har ingen konsekvenser for den behandling der tilbydes at takke nej til projektet. Ved accept underskrives samtykkeerklæringen. Samtykket kan på et hvilket som helst tidspunkt trækkes tilbage uden det får indflydelse på barnets behandling. Oplysninger videregives således kun fra kommunerne til Enheden for Overvægtige Børn og Unge såfremt der er skriftligt samtykke hertil fra den enkelte familie .

**Projektets aktører**

1. Enheden for Overvægtige Børn og Unge/Den Danske Biobank for Overvægtige Børn, Børneafdelingen, Holbæk Sygehus.

2. Kommuner i Region Sjælland: Kalundborg, Holbæk, Stevns og Slagelse.

3. Kommuner uden for Region Sjælland: Hedensted, Horsens, Kolding og Vejle.

**Praktisk organisering**

Enheden for Overvægtige børn og unge forestår oplæring, supervisionen og løbende undervisning af de otte kommuners behandlere. De forestår ligeledes den overordnede data indsamling og analyse. Hertil er særligt allokeret en ph.d. studerende, en projektsygeplejerske på deltid (26 timer) og en bioanalytiker på deltid (26 timer). Hver enkelt kommune forestår de ansættelse af behandlere og lokal drift.

**Databehandling og evaluering**

Data fra alle undersøgelser og skemaer indtastes løbende i en allerede eksisterende og omfattende database i Enheden for Overvægtige Børn.

**Formidling**

Projektets resultater forventes offentliggjort i regionale, nationale og internationale aviser og tidsskrifter, samt udfærdiges til en ph.d.-afhandling.

# Anmeldelse

Projektet indgår i Den Danske Biobank for Overvægtige Børn, som er godkendt af Region Sjællands Videnskabsetiske Komité med protokol SJ-104. Biobanken anmeldt og godkendt af Datatilsynet og registreret på Clinicaltrial.gov med ID-no. NCT00928473, ligesom aktuelle studie særskilt er registreret med ID-no. NCT02013843.

**Tidsramme**

Startdato: 1.10.12

Slutdato: 28.2.16.

Etableringsfase: Oktober 2012 – Februar 2013

Etablering af samarbejde og oplæring af kommunalt personale

Udarbejdelse af skemaer og informationsmateriale til kommunalt brug

Inklusion og behandling: Marts 2013 – Marts 2015.

Behandling af 100 børn i hver kommune, med løbende supervision lokalt og central undervisning

Løbende data indsamling og indtastning i database

Analyse og resultatbehandling:

April 2015- Marts 2016

Analyse og udarbejdelse af ph.d.-afhandling

**Budget**

Projektet er finansieret med 2,6 mio. kr. fra Region Sjælland, dækkende.

Den resterende del søges, og er delvist finansieret ved Regions Sjælland Sundhedsvidenskabelige Forskningsfond, samt af en række andre private fonde og legater, som ansøges løbende.

**Referencer**

1. Pearson S, Olsen LW, Hansen B, Sørensen TIA. [Increase in overweight and obesity amongst Copenhagen schoolchildren, 1947-2003]. Ugeskr Laeger. 2005;167: 158–162. Available: http://www.ncbi.nlm.nih.gov/pubmed/15697126

2. Pearson S, Hansen B, Sørensen TI, Baker JL. Overweight and obesity trends in Copenhagen schoolchildren from 2002 to 2007. Acta Paediatr (Oslo, Norw 1992). 2010;99: 1675–1678. doi:10.1111/j.1651-2227.2010.01897.x

3. Wang Y, Lobstein T. Worldwide trends in childhood overweight and obesity. Int J Pediatr Obes. 2006;1: 11–25. doi:10.1080/17477160600586747

4. Jackson-Leach R, Lobstein T. Estimated burden of paediatric obesity and co-morbidities in Europe. Part 1. The increase in the prevalence of child obesity in Europe is itself increasing. Int J Pediatr Obes. 2006;1: 26–32. doi:10.1080/17477160600586614

5. Grønbæk HN, Holm J-C. [Psychological consequences of severe overweight in teenagers]. Ugeskr Laeger. 2011;173: 1785–1791. Available: http://www.ncbi.nlm.nih.gov/pubmed/21689505

6. Holm J-C, Gamborg M, Neland M, Ward L, Gammeltoft S, Heitmann BL, et al. Longitudinal changes in blood pressure during weight loss and regain of weight in obese boys and girls. J Hypertens. 2012;30: 368–374. doi:10.1097/HJH.0b013e32834e4a87

7. Nielsen TRH, Gamborg M, Fonvig CE, Kloppenborg J, Hvidt KN, Ibsen H, et al. Changes in lipidemia during chronic care treatment of childhood obesity. Child Obes. 2012;8: 533–41. doi:10.1089/chi.2011.0098

8. Fonvig C, BIlle D, Chabanova E, Nielsen T, Holm J-C. Muscle fat content and abdominal adipose tissue distribution investigated by magnetic resonance spectroscopy and imaging in obese children and youths. Pediatr Rep. 2012;2012: e11. Available: http://www.ncbi.nlm.nih.gov/pmc/articles/PMC3357610/

9. Bille DS, Chabanova E, Gamborg M, Fonvig CE, Nielsen TRH, Thisted E, et al. Liver fat content investigated by magnetic resonance spectroscopy in obese children and youths included in multidisciplinary treatment. Clin Obes. 2012;2: 41–49. doi:10.1111/j.1758-8111.2012.00038.x

10. Oude Luttikhuis H, Baur L, Jansen H, Shrewsbury VA, O’Malley C, Stolk RP, et al. Interventions for treating obesity in children. Cochrane Libr. 2009; CD001872. doi:10.1002/14651858.CD001872.pub2

11. Dolinsky DH, Armstrong SC, Walter EB, Kemper AR. The effectiveness of a primary care-based pediatric obesity program. Clin Pediatr (Phila). 2012;51: 345–53. doi:10.1177/0009922811425232

12. Spear BA, Barlow SE, Ervin C, Ludwig DS, Saelens BE, Schetzina KE, et al. Recommendations for Treatment of Child and Adolescent Overweight and Obesity. Pediatrics. 2007;120: S254–S288. doi:10.1542/peds.2007-2329F

13. Gerner B, McCallum Z, Sheehan J, Harris C, Wake M. Are general practitioners equipped to detect child overweight/obesity? Survey and audit. J Paediatr Child Health. 2006;42: 206–211. doi:10.1111/j.1440-1754.2006.00831.x

14. Cretikos M, Valenti L, Britt H, Baur L. General practice management of overweight and obesity in children and adolescents in Australia. Med Care. 2008;46: 1163–1169. doi:10.1097/MLR.0b013e318179259a

15. Saelens BEB, Sallis JJF, Wilfley DE, Patrick K, Cella JA, Buchta R. Behavioral weight control for overweight adolescents initiated in primary care. Obes Res. 2002;10: 22–32. doi:10.1038/oby.2002.4

16. O’Connor TM, Hilmers A, Watson K, Baranowski T, Giardino AP. Feasibility of an obesity intervention for paediatric primary care targeting parenting and children: Helping HAND. Child Care Health Dev. 2013;39: 141–149. doi:10.1111/j.1365-2214.2011.01344.x

17. Robertson W, Thorogood M, Inglis N, Grainger C, Stewart-Brown S. Two-year follow-up of the Families for Health programme for the treatment of childhood obesity. Child Care Heal Dev. 2012;38: 229–36. doi:10.1111/j.1365-2214.2011.01237.x

18. Robertson W, Stewart-brown S, Stallard N, Petrou S, Griffiths F, Thorogood M, et al. Evaluation of the effectiveness and cost-effectiveness of Families for Health V2 for the treatment of childhood obesity : study protocol for a randomized controlled trial. Trials. Trials; 2013;14: 81. doi:10.1186/1745-6215-14-81

19. Smith LR, Chadwick P, Radley D, Kolotourou M, Gammon CS, Rosborough J, et al. Assessing the short-term outcomes of a community-based intervention for overweight and obese children: The MEND 5-7 programme. BMJ Open. 2013;3: 1–7. doi:10.1136/bmjopen-2013-002607

20. Holm J-C, Gamborg M, Bille DS, Grønbæk HN, Ward LC, Faerk J. Chronic care treatment of obese children and adolescents. Int J Pediatr Obes. 2011;6: 188–196. doi:10.3109/17477166.2011.575157

**Oversigt over bilag.**

1. Godkendelse fra Videnskabsetisk komite – SJ-104. Den Danske Biobank for Overvægtige Børn.
2. Godkendelse af tillægsprotokol nr. 10 til SJ-104. Vedr. aktuelle projekt.
3. Godkendelse fra Datatilsynet.
4. Bevillingsaftale fra Region Sjælland.

**Bilag. 1 Godkendelse fra Videnskabsetisk komité**

**– SJ-104 (Den Danske Biobank for Overvægtige Børn)**

**Bilag 2. Godkendelse af tillægsprotokol nr. 10 til SJ-104.**

**Vedr. aktiviteterne i aktuelle projekt.**

**Bilag 3. Godkendelse fra Datatilsynet.**

**Bilag 4. Bevillingsaftale fra Region Sjælland.**
